# Impact of LITAF on Mitophagy and Neuronal Damage in Epilepsy via MCL‐1 Ubiquitination

**DOI:** 10.1111/cns.70191

**Published:** 2025-01-07

**Authors:** Fuli Min, Zhaofei Dong, Shuisheng Zhong, Ze Li, Hong Wu, Sai Zhang, Linming Zhang, Tao Zeng

**Affiliations:** ^1^ Department of Neurology, School of Medicine, Guangzhou First People's Hospital South China University of Technology Guangzhou China; ^2^ Department of Neurology, the Eighth Affiliated Hospital Sun Yat‐Sen University Shenzhen China; ^3^ Department of Neurology Guangdong Sanjiu Brain Hospital Guangzhou China; ^4^ Department of Neurology The First Affliated Hospital of Kunming Medical University Kunming China; ^5^ Department of Neurology, Zhujiang Hospital Southern Medical University Guangzhou China

**Keywords:** epilepsy, LPS‐induced TNF‐alpha factor, MCL1, mitochondrial autophagy, neuroprotection, ubiquitination regulation

## Abstract

**Objective:**

This study aims to investigate how the E3 ubiquitin ligase LITAF influences mitochondrial autophagy by modulating MCL‐1 ubiquitination, and its role in the development of epilepsy.

**Methods:**

Employing single‐cell RNA sequencing (scRNA‐seq) to analyze brain tissue from epilepsy patients, along with high‐throughput transcriptomics, we identified changes in gene expression. This was complemented by in vivo and in vitro experiments, including protein–protein interaction (PPI) network analysis, western blotting, and behavioral assessments in mouse models.

**Results:**

Neuronal cells in epilepsy patients exhibited significant gene expression alterations, with increased activity in apoptosis‐related pathways and decreased activity in neurotransmitter‐related pathways. LITAF was identified as a key upregulated factor, inhibiting mitochondrial autophagy by promoting MCL‐1 ubiquitination, leading to increased neuronal damage. Knockdown experiments in mouse models further confirmed that LITAF facilitates MCL‐1 ubiquitination, aggravating neuronal injury.

**Conclusion:**

Our findings demonstrate that LITAF regulates MCL‐1 ubiquitination, significantly impacting mitochondrial autophagy and contributing to neuronal damage in epilepsy. Targeting LITAF and its downstream mechanisms may offer a promising therapeutic strategy for managing epilepsy.

## Graphical abstract

The study reveals that the E3 ubiquitin ligase LITAF promotes MCL‐1 ubiquitination, impairing mitophagy and exacerbating oxidative stress‐induced neuronal damage in epilepsy. Targeting LITAF and its regulation of MCL‐1 may offer a novel therapeutic strategy for mitigating neuronal damage and treating epilepsy.

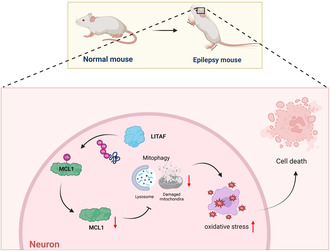

## 1 Introduction

Epilepsy (EP) is a prevalent neurological disorder characterized by recurrent episodes of temporary brain dysfunction [1, 2, 3, 4]. These episodes are triggered by excessive neuronal discharges [5]. The causes of EP are multifaceted, ranging from genetic predispositions to brain injuries and encephalitis; however, the precise molecular mechanisms underlying the condition remain an area of critical research [2, 3, 4, 6]. To gain deeper insights into the pathological mechanisms of EP and identify potential therapeutic strategies, we have focused on exploring the interaction between the E3 ubiquitin ligase and MCL‐1, and their regulation of mitochondrial autophagy.

Mitophagy is the primary cellular mechanism for clearing damaged mitochondria, which is essential for maintaining cellular homeostasis and functionality [7, 8, 9]. Recent studies have linked mitophagy dysregulation to the frequency and severity of EP seizures [10, 11, 12]. MCL‐1, a well‐established regulator of apoptosis, has recently been implicated in the regulation of mitophagy [13, 14, 15, 16]. The stability and regulation of MCL‐1 are crucial for cell survival, particularly in managing cellular stress and promoting neuroprotection [17, 18]. Recent research has revealed the critical role of the ubiquitination (Ub) process of MCL‐1 in regulating neuronal survival [19], offering a new perspective for treating neurologic diseases associated with cell death.

Ubiquitination is a posttranslational modification process that involves the attachment of the small protein ubiquitin to target proteins, typically leading to the degradation of the target protein [20]. E3 ubiquitin ligases are key enzymes in the Ub pathway, capable of selectively mediating Ub for specific target proteins [21]. Recent studies have demonstrated that E3 ubiquitin ligases play crucial roles in various neurological disorders, with important implications for neuroprotection and mitochondrial quality control [22, 23]. Considering the function of MCL‐1 and its role in mitophagy, we hypothesize that E3 ubiquitin ligases may regulate its function and mitophagy activity by mediating MCL‐1 Ub.

LPS‐induced TNF‐alpha factor (LITAF) is a known E3 ubiquitin ligase, and recent studies suggest its involvement in the pathogenesis of various neurological disorders [24, 25, 26]. Currently, there is limited understanding of the role of E3 ubiquitin ligases, particularly LITAF, in regulating MCL‐1 Ub and mitophagy, as well as how this process specifically impacts the mechanisms underlying EP. Therefore, this study proposes an innovative approach that combines bioinformatics and experimental biology to investigate the role of E3 ubiquitin ligases, especially LITAF, in regulating MCL‐1 Ub and mitophagy and further explore how these mechanisms operate in the pathogenesis of EP.

The uniqueness of this research strategy lies in its integration of high‐throughput bioinformatics analysis and precise molecular biology techniques, enabling a comprehensive understanding of the role of E3 ubiquitin ligases in neurological disorders from a systems biology perspective. Building upon the cutting‐edge knowledge and background outlined above, our study aims to gain deeper insights into the interaction between LITAF and MCL‐1 and their impact on mitophagy, shedding light on how this process contributes to the onset and progression of EP. This research not only promises to provide a novel theoretical foundation for understanding the molecular mechanisms of EP but also holds the potential to identify new therapeutic targets for EP in the future.

## 2 Materials and Methods

### 2.1 Acquisition of Single‐Cell RNA Sequencing (scRNA‐Seq) Data Related to EP

Single‐cell RNA sequencing data related to EP were obtained from the Gene Expression Omnibus (GEO) database (https://www.ncbi.nlm.nih.gov/geo/) under accession number GSE190452. Two healthy control samples (GSM5724021 and GSM5724022) and two EP patient samples (GSM5724026 and GSM6893725) were selected from the dataset for analysis using the R software package “Seurat” [27]. Data quality control was conducted using criteria of 200 < nFeature_RNA < 5000 and percent.mt < 25, followed by a selection of the top 2000 highly variable genes based on variance [28].

### 2.2 UMAP Clustering Analysis and Cell Annotation

To reduce the dimensionality of the scRNA‐seq dataset, principal component analysis (PCA) was performed on the highly variable genes, selecting the top 2000 genes with the highest variance. The top 30 principal components were used for downstream analysis via the Elbowplot function in Seurat. The FindClusters function identified major cell subclusters (resolution = 1). The dataset was then processed with UMAP for nonlinear dimensionality reduction. Cell annotation was conducted by integrating known cell lineage‐specific marker genes and using the online tool CellMarker, with marker genes for each cell type provided in Table S1 [29].

### 2.3 RNA Extraction and Sequencing

Normal mice were injected with kainic acid (KA; HY‐N2309, MedChemExpress). Hippocampal tissues were harvested at 6 and 12 h post‐injection from EP model mice (three samples per time point). Total RNA was extracted using Trizol reagent (15,596,026, Invitrogen, Carlsbad, CA, USA) and quantified using a Nanodrop 2000 spectrophotometer (1011 U, Nanodrop, USA). Samples with an RNA Integrity Number (RIN) ≥ 7.0 and a 28S:18S ratio ≥ 1.5 were included. Sequencing libraries were prepared using the Ribo‐Zero Magnetic Kit (MRZE706, Epicentre Technologies) and the NEB Next Ultra RNA Library Prep Kit (#E7775, NEB, USA) for Illumina sequencing [30].

The sequencing libraries were generated and sequenced by CapitalBio Technology (Beijing, China). Each sample utilized a total of 5 μg of RNA. Briefly, the Ribo‐Zero Magnetic Kit (MRZE706, Epicentre Technologies, Madison, WI, USA) was employed to deplete ribosomal RNA (rRNA) from the total RNA. The NEB Next Ultra RNA Library Prep Kit (#E7775, NEB, USA) was used for Illumina library construction and preparation for sequencing. Subsequently, RNA fragments were size‐selected into approximately 300 base pair (bp) fragments using NEB Next First Strand Synthesis Reaction Buffer (5×).

The first cDNA strand was synthesized using reverse transcriptase primers and random primers, followed by the second cDNA strand synthesis in Second Strand Synthesis Reaction Buffer supplemented with dUTP Mix (10×). The cDNA fragments underwent end repair, including polyA tail addition and sequencing adapter ligation. After attaching Illumina sequencing adapters, the second cDNA strand of the cDNA was digested using USER Enzyme (#M5508, NEB, USA) to construct a strand‐specific library. The library DNA was then amplified, purified, and enriched via PCR. Subsequently, library identification was performed using the Agilent 2100 Bioanalyzer, and quantification was carried out with the KAPA Library Quantification Kit (KK4844, KAPA Biosystems). Finally, paired‐end sequencing was conducted on the NextSeq CN500 (Illumina) sequencer platform [30].

### 2.4 Quality Control of Sequencing Data and Alignment to the Reference Genome

The quality of paired‐end reads in the raw sequencing data was assessed using FastQC software version 0.11.8. Preprocessing of the raw data was conducted with Cutadapt software version 1.18 to eliminate Illumina sequencing adapters and poly(A) tail sequences. A perl script was employed to filter out reads with N content exceeding 5%. Reads with a base quality of at least 20 and constituting 70% of the total bases were extracted using FASTX Toolkit software version 0.0.13. BBMap software was utilized to repair paired‐end sequences. Subsequently, the filtered high‐quality read fragments were aligned to the mouse reference genome using HISAT2 software version 0.7.12 [30].

### 2.5 Differential Gene Analysis

The “limma” package in R software [31] was utilized to conduct differential gene expression analysis on the sequencing data, employing |logFC| > 1 and Padjust < 0.05 as the selection criteria for the analysis of differentially expressed genes. Subsequently, visualization of the differential analysis results was performed using the “ggplot2” package in R to create relevant volcano plots [32].

The “VennDiagram” package in R was employed to conduct an intersection analysis of the obtained differentially expressed genes and genes associated with mitophagy, followed by the generation of a Venn diagram illustrating the intersecting genes [33]. The genes related to mitophagy were sourced from the GeneCards database (https://www.genecards.org/).

### 2.6 GO Enrichment Analysis

Differentially expressed genes were analyzed for Gene Ontology (GO) enrichment using the “clusterProfiler,” “org.Mm.eg.db,” “enrichplot,” and “ggplot2” packages in R. Bubble plots were generated with enrichplot to visualize the enrichment results for the biological process (BP), cellular component (CC), and molecular function (MF) categories within the GO framework [34].

### 2.7 Construction of Protein–Protein Interaction (PPI) Network and Prediction of Target Genes

The STRING database, which integrates experimental data, text mining results from PubMed abstracts, and other sources, was used to explore protein interactions. To predict interactions among 11 mitophagy‐related factors significantly differentially expressed in the early stages of epilepsy, we used STRING (http://www.string‐db.org/), with the species set to mouse and a confidence threshold of 0.4 [35].

### 2.8 Ubibrowser Database

The online Ubibrowser database (http://ubibrowser.bio‐it.cn/) was used to predict the E3 ubiquitin ligase associated with MCL1 ubiquitination. Predictions were made with a confidence interval ranging from 0.825 to 0.896 [36].

### 2.9 Establishment of EP Mouse Model

Sixty eight‐week‐old C57BL/6N mice were purchased from Beijing Vital River Laboratory Animal Technology Co. Ltd. (213, Beijing, China). The mice were housed in standard cages with a constant room temperature of 23°C ± 1°C, a 12‐h light/dark cycle, and ad libitum access to food and water. A 1‐week acclimatization period was conducted prior to the experiments. The experimental procedures and animal handling protocols were approved by the Institutional Animal Ethics Committee of The Eighth Affiliated Hospital, Sun Yat‐Sen University (approved number: SYSU‐IACUC‐2020‐B0178).

The mice were anesthetized with chloral hydrate (400 mg/kg, YT0533, ItaBio, China) and administered with 50 nanoliters of a solution containing 20 millimoles of KA (Sigma‐K0250, St. Louis, MO) unilaterally in the dorsal hippocampus using stereotaxic injection (Figure S1), or with 50 nanoliters of 0.9% NaCl solution (saline) on the same side. The injection coordinates were as follows: anteroposterior (AP)‐2, mediolateral (ML)‐1.5, dorsoventral (DV)‐2, with the reference point being the bregma on the mouse skull. Following recovery from anesthesia (approximately 2 h), the mice underwent a visual examination to observe their behavior during the sustained EP state induced by KA. Mice injected with KA into the hippocampus exhibited mild asymmetrical rigid movements of the forelimbs, rigid head deviation, spinning, and/or prolonged immobility, often accompanied by bilateral forelimb spasms and hindlimb standing. Only mice displaying this characteristic behavioral pattern were included in the subsequent analysis stage [37].

The shRNA (short hairpin RNA) vector is transcribed into pre‐shRNA (primary shRNA) within the cell nucleus. It is then processed into pre‐shRNA (precursor shRNA) by the double‐stranded RNA‐binding proteins DGCR8 and Drosha. The pre‐shRNA is transported from the nucleus to the cytoplasm with the assistance of protein factors such as Exportin‐5. In the cytoplasm, the pre‐shRNA is processed by the Dicer enzyme and other protein factors, removing its stem‐loop structure to form siRNA, thereby initiating the RNAi process to silence the expression of the target gene. Based on this principle, shRNA knockdown vectors targeting specific sequences were designed to induce RNA‐specific degradation [38].

The core plasmid pLL3.7‐Fgf2 containing the target gene silencing sequence, along with the helper plasmids (psPAX2 and pMD2.G), were used to package the silencing lentivirus. The plasmid and lentivirus packaging services were provided by Gene Tech Bioengineering (Shanghai, China). Mcl1 or Litaf knockdown mice were constructed via lentiviral injection, with the sham‐operated group receiving the same dose of empty lentiviral vector at a viral concentration of 5 × 10^6^ TU/mL. Before the experiments, the mice were acclimated to these conditions for 2–3 days, then anesthetized and placed into a stereotaxic apparatus. One microliter of lentivirus was injected into the DG/CA1 region of the mouse hippocampus (anterior/posterior: −2.0 mm, medial/lateral: ±1.5 mm, dorsal/ventral: −2.0/2.5 mm). The viral injection was performed using a 5 μL Hamilton syringe (Hamilton‐87,900, Reno, USA) at a rate of 0.05 μL/min. After the injection, the syringe was left in place for 10 min to minimize reflux at the injection site. The same procedure was carried out on the contralateral hippocampal region [39].

The expression levels of Mcl1 and Litaf in lentivirus‐transfected cells were detected by RT‐qPCR and Western Blot, and the sequences with the highest silencing efficiency were selected for subsequent experiments.

The animal experimental groups were as follows: Normal group (normal mice injected with saline), EP group (epileptic mice injected with KA), Control + sh‐NC group (normal mice injected with saline + lentiviral control sequence), EP + sh‐NC group (KA‐injected mice + lentiviral control sequence), EP + sh‐Mcl1 group (KA‐injected mice + Mcl1 silencing lentivirus), EP + sh‐Litaf group (KA‐injected mice + Litaf silencing lentivirus). Each group contained eight mice (*n* = 8). The silencing transfection sequences are provided in Table 1.

**TABLE 1:** Lentivirus transfection primer sequence.

Name	Sequence
sh‐Mcl1‐1 (Mouse)	5′‐CCAAACACTTAAAGAGCGTAA‐3′
sh‐Mcl1‐2 (Mouse)	5′‐GCAGGATTGTGACTCTTATTT‐3′
sh‐Litaf‐1 (Mouse)	5′‐CCACTCAAGAACTGTCAAGTA‐3′
sh‐Litaf‐2 (Mouse)	5′‐ATTGCCGTGCAGACCGTTTAT‐3′
sh‐NC	5′‐CCTAAGGTTAAGTCGCCCTCG‐3′

### 2.10 Behavioral Monitoring of Epileptic Seizures and Local Field Potential (LFP) Detection

Following the successful establishment of the EP model in mice, spontaneous recurrent seizures (SRS) were monitored behaviorally for 24 h. One month after EP induction, LFP recordings were performed. The procedure involved anesthetizing the mice, exposing the skull, and securing them in a stereotaxic apparatus. Using the xyphoid process as a reference, micropositioning was conducted by drilling holes 2.0 mm posterior and 1.5 mm lateral to the midline. Two stainless steel screws were inserted into the frontal bone for grounding, and an electrode array was fixed to the stereotaxic frame. The electrodes were lowered 1.5 mm into the hole and secured with dental cement. Sterile cotton was used to manage hemostasis during drilling. To prevent excessive movements, the mice were immobilized during LFP fluctuations. LFP signals were recorded using the MAP data acquisition system (Plexon, Dallas, TX), filtered (0.1–500 Hz), preamplified (1000×), and digitized at 4 kHz. The data were analyzed using NeuroExplorer software (Nex Technologies, Littleton, MA). Seizure‐like events (SLE) were defined as spontaneous bursts of activity lasting 5 s or more, with peak amplitudes at least twice the standard deviation (SD) and frequencies greater than 1 Hz. Subsequent analyses, such as Western blotting and imaging, were performed on the same animals used in behavioral experiments [39, 40, 41].

### 2.11 Neuronal Cell Isolation and Culture

The day before cell isolation, culture plates, bottles, or dishes were coated with 0.01% poly‐L‐lysine and incubated overnight. The next morning, the coating solution was removed, the dishes air‐dried in a sterile laminar flow hood and then placed in an incubator for later use. A specialized neuronal culture medium was prepared, consisting of 1% glutamine in DMEM/F12 medium (11320033), 2% B27 (A1486701), and 1% antibiotics (15070063), all purchased from Thermo Fisher (USA). EP rats and normal mice were sacrificed 12 h post‐injection, disinfected with 75% alcohol, and the scalp and skull were aseptically removed to expose the brain. The brain was washed with precooled PBS, and the crescent‐shaped hippocampal formation was carefully extracted. Microvessels were meticulously removed, and the tissue was minced using ophthalmic scissors. The tissue was then incubated with 0.125% trypsin for 30 min, with gentle shaking every 5 min. The digestion was stopped by adding fetal bovine serum, and the cell suspension was gently pipetted until it became viscous. The suspension was filtered using a 200‐mesh cell strainer, centrifuged at 1000 rpm for 5 min, and the cells resuspended in DMEM/F12 complete medium. The cell concentration was adjusted to 100,000 cells/mL and seeded into precoated dishes. After 6 h, the medium was replaced with a specialized neuronal culture medium. At 48 h, ara‐C was added to inhibit the growth of non‐neuronal cells, and half of the media was replaced every 3 days. The purity of the neuronal cells was confirmed by Neuronal Nuclei (NeuN) immunofluorescence staining [42].

### 2.12 Cell Transfection

For lentivirus‐mediated transfection, 5 × 10^5^ cells were seeded into a 6‐well plate and cultured until 70%–90% confluence was reached. Cells were then transfected with the appropriate dose of packaged lentivirus (MOI = 10, with a working titer of approximately 5 × 10^6^ TU/mL) and 5 μg/mL polybrene (Merck, TR‐1003, USA). After 4 h, an equal volume of fresh medium was added to dilute the polybrene, and after 24 h, the medium was replaced with fresh medium. Transfection efficiency was evaluated 48 h post‐transfection using a luciferase reporter gene, and stably transfected cell lines were selected by adding 1 μg/mL puromycin (Thermo Fisher, A1113803, USA) [43, 44, 45]. RNA was isolated to assess infection efficiency via RT‐qPCR, and sequences with good selection efficiency were used for subsequent experiments [46]. Each experiment was repeated three times. Recombinant lentivirus and plasmids were provided by Shanghai GenEngine BioTech Co. Ltd. (Shanghai, China), and the silencing transfection sequences are listed in Table 1.

The cell groups used in the study were as follows: Control (normal mouse neuronal cells transfected with silencing control sequence), sh‐NC (normal mouse neuronal cells transfected with silencing control sequence), sh‐Mcl1 (normal mouse neuronal cells transfected with Mcl1 silencing sequence), EP + sh‐NC (epileptic mouse neuronal cells transfected with silencing control sequence), EP + sh‐Litaf (epileptic mouse neuronal cells transfected with Litaf silencing sequence), and EP + sh‐Litaf + sh‐Mcl1 (epileptic mouse neuronal cells transfected with both Litaf and Mcl1 silencing sequences).

### 2.13 Western Blot Experiment

Total proteins from cells and tissues were extracted using a protein extraction kit (Bestbio, BB3101, Shanghai, China), and the concentration was measured using a BCA assay kit (Beyotime, P0012S, Shanghai, China). Samples (50 μg) were loaded onto a 10% SDS‐PAGE gel (Beyotime, P0012A, Shanghai, China) and electrophoresed at a constant voltage of 80–120 V for 2 h. Proteins were transferred onto a PVDF membrane (Merck, IPVH00010, Germany) at a constant current of 250 mA for 90 min under wet conditions. The membrane was blocked with TBST containing 5% skim milk at room temperature for 2 h, followed by three washes in TBST for 10 min each. Primary antibodies (listed in Table 2) were incubated overnight at 4°C, and the membrane was washed three times in TBST. The secondary antibodies, goat anti‐rabbit IgG (1:2000, Abcam, ab6721, UK) or goat anti‐mouse IgG (1:2000, Abcam, ab6789, UK), were applied for 1 h at room temperature, followed by three 10‐min washes in PBST. The ECL substrate (Beyotime, P0018FS, Shanghai, China) was used for visualization, and images were developed in a darkroom [47]. All samples were analyzed in triplicate.

**TABLE 2:** Western blot antibody information.

Target name	Manufacturer	Article number	Dilution ratio
MCL1 (Mouse)	Thermofisherr	MA5‐32060	1:1000
LITAF (Mouse)	Thermofisherr	PA5‐96333	1:1000
PINK1 (Mouse)	Thermofisherr	PA1‐16604	1:500
LC3‐I/LC3‐II (Mouse)	Thermofisherr	PA1‐16931	1:500
TIMM23 (Mouse)	Thermofisherr	MA5‐27384	1:1000
TOMM20 (Mouse)	Thermofisherr	PA5‐52843	1:250
GAPDH (Mouse)	Thermofisherr	MA5‐15738	1:1000

*Note:* Thermofisher, USA.

### 2.14 Detection of MCL1 Ub Levels

Mouse hippocampal tissue or neuronal cells were lysed using a buffer containing HEPES (15630106, Thermo Fisher Scientific, USA), consisting of 20 mM HEPES (pH 7.2), 50 mM NaCl, 1 mM NaF, 0.5% Triton X‐100, 0.1% SDS, 20 μM MG132 (133407‐82‐6, Thermo Fisher Scientific, USA), and a protease inhibitor (87785, Thermo Fisher Scientific, USA). Lysates were incubated overnight with anti‐MCL1 antibody (MA5‐32060, Thermo Fisher Scientific, 1:500), followed by a 4‐h incubation with Protein A/G agarose beads (78610, Thermo Fisher Scientific, USA) to isolate MCL1 protein. The beads were washed three times with HEPES buffer to remove nonspecific proteins. The bound protein was released by heating the beads in SDS‐PAGE buffer, followed by immunoblotting. The intensity of the ubiquitinated MCL1 protein bands was analyzed to assess changes in MCL1 ubiquitination levels [48].

### 2.15 RT‐qPCR

Total RNA was extracted using Trizol (catalog no. 16096020, Thermofisher, USA), and the concentration and purity were evaluated using the NanoDrop One/OneC analyzer (Thermofisher), with an A260/A280 ratio of 2.0 and a concentration greater than 5 μg/μL. cDNA synthesis was performed using the cDNA First Strand Synthesis Kit (catalog no. D7168L, Beyotime, Shanghai, China). The RT‐qPCR kit (catalog no. Q511‐02, Vazyme Biotech, Jiangsu, China) was used according to the manufacturer's instructions. The reaction mixture included 2 μL of cDNA template, 0.2 μL of each primer, and 10 μL of RT‐qPCR Mix, adjusted to a total volume of 20 μL with RNAase‐free water. PCR amplification was performed using the Bio‐Rad CFX96 system under the following conditions: initial denaturation at 95°C for 30 s, followed by 40 cycles of denaturation at 95°C for 10 s, annealing at 60°C for 30 s, and extension at 72°C for 30 s. A melt curve analysis was conducted from 65°C to 95°C. Primer sequences were obtained from Shanghai Sangon Biotech (Shanghai, China) and are listed in Table 3. Gene expression was calculated using the 2^−ΔΔCt^ method with β‐actin as the reference gene. All experiments were performed in triplicate [49, 50].

**TABLE 3:** RT‐PCR primer sequence.

Gene	Primer sequence(5′–3′)
MCL1 (Mouse)	F: AAAGGCGGCTGCATAAGTC
	R: TGGCGGTATAGGTCGTCCTC
LITAF (Mouse)	F: ATGTCGGCTCCAGGACCTTA
	R: GGTAGTAACTGTTGACACCCACT
GAPDH (Mouse)	F: AGGTCGGTGTGAACGGATTTG
	R: GGGGTCGTTGATGGCAACA

### 2.16 Detection of Reactive Oxygen Species (ROS) Using DCFH‐DA Method

Intracellular ROS levels were measured using 2′,7′‐dichlorofluorescein diacetate (DCFH‐DA) (C400, Thermo Fisher, USA). DCFH is oxidized by ROS into the highly fluorescent compound DCF. Neurons in a 96‐well plate were washed with PBS and incubated with DCFH‐DA (10 μM) in the dark for 30 min. Fluorescence intensity was measured with a fluorescence microplate reader (SpectraMax Gemini EM, Molecular Devices, Sunnyvale, CA, USA) at excitation/emission wavelengths of 485 nm/535 nm. Cell images were captured with a laser scanning confocal microscope (FV1000, Olympus, Japan) and analyzed using FV10‐ASW 4.0 software (Olympus) [51].

### 2.17 Immunohistochemistry

Mouse brain tissues were embedded in paraffin, sectioned, and baked at 60°C for 20 min. The sections were immersed in xylene, dehydrated in alcohol, and rehydrated. Endogenous peroxidase activity was blocked with 3% H_2_O_2_ for 10 min. Antigen retrieval was achieved using citrate buffer and microwaving. Sections were washed with PBS and blocked with normal goat serum (E510009, Shanghai Shenggong Bioengineering Co. Ltd.) for 20 min at room temperature. Primary antibodies (Mcl‐1: MA5‐32060, Litaf: PA5‐96333, ThermoFisher) were incubated overnight at 4°C. After washing, sections were incubated with a secondary antibody (goat anti‐rabbit IgG, ab6721, 1:500, Abcam, UK) for 30 min. Sections were then stained with DAB chromogen reagent (DAB150, Sigma, USA) and counterstained with hematoxylin for 30 s. After sequential dehydration, sections were cleared in xylene and mounted with neutral resin. The stained sections were observed with a brightfield microscope (BX63, Olympus, Japan). Immunostaining was analyzed using the Aperio Scanscope System (Vista, CA) to quantify protein expression based on the total protein‐positive area and tissue weight [52].

### 2.18 Immunofluorescence Staining

Cells were seeded onto a 12‐well plate a day before the experiment. After the cells adhered, they were washed with PBS and fixed with 4% paraformaldehyde (PFA). Cells were permeabilized with 0.1% Triton X‐100, blocked with 10% donkey serum, and incubated overnight at 4°C with NeuN antibody (PA5‐78499, Thermo Fisher, USA; 1:250). On the following day, cells were washed and incubated with Alexa Fluor 555‐conjugated goat anti‐mouse IgG (A‐21137, Thermo Fisher, USA) for 1 h at room temperature. After staining the nuclei with DAPI (C1002, Beyotime, China), the slides were mounted with anti‐fade fluorescence mounting medium and observed under a fluorescence microscope (FV‐1000/ES, Olympus, Japan). Fluorescence coverage was quantified using a 40× objective lens and six fixed fields per group [53].

### 2.19 Investigation of Mitophagy Using Co‐Focused Immunofluorescence Microscopy

Neuronal cells were pretreated and stained with 400 nM MitoTracker Deep Red (M22426, Invitrogen, USA) for 30 min at 37°C. After staining, cells were fixed with 4% PFA and permeabilized with 0.2% Triton X‐100. Cells were blocked with 1% BSA in PBS and incubated overnight at 4°C with an anti‐LC3 antibody (PA1‐46286, Invitrogen, USA). After washing, cells were incubated with Alexa Fluor 488‐conjugated goat anti‐rabbit antibody (A‐11008, Invitrogen, USA), followed by nuclear staining with DAPI (300 nM; D1306, Invitrogen, USA). Cells were mounted with ProLong Gold Antifade Mountant (P36930, Invitrogen, USA) and imaged using an LSM 700 microscope with a 63×/1.4 NA objective lens (Carl Zeiss Microscopy, Germany) [54].

### 2.20 TUNEL Staining

Neuronal cells were fixed with 4% PFA and permeabilized with 0.25% Triton X‐100. Cells were blocked with 5% bovine serum albumin (BSA, Yeasen, China), followed by incubation with TUNEL reagent (C1086, Beyotime, China). After staining with DAPI (C1002, Beyotime), cells were imaged using a confocal microscope (LSM 880, Carl Zeiss AG, Germany). TUNEL‐positive cells (green fluorescence) were identified as apoptotic, while DAPI‐stained nuclei (blue fluorescence) represented total cell counts. Apoptosis rates were calculated as the percentage of apoptotic cells among total cells [55].

### 2.21 Levels of Oxidative Stress

Malondialdehyde (MDA) levels were determined using a Thiobarbituric Acid Reactive Substances assay kit (S0131S, Beyotime, China). Cells or tissues were mixed with 0.2 mM MDA detection solution, heated at 100°C for 15 min, and centrifuged. The absorbance of the supernatant was measured at 532 nm to calculate MDA levels. Superoxide dismutase (SOD) levels were measured using a separate assay kit (S0088, Beyotime, China) according to the manufacturer's protocol, with absorbance measured at 560 and 412 nm [56].

### 2.22 Observations Under Transmission Electron Microscope

Hippocampal tissue slices were fixed in 3% glutaraldehyde for 1 h, followed by fixation in 1% osmium tetroxide for 3 h. The slices were then washed three times with 0.1 mol/L phosphate buffer at pH 7.4, each wash lasting 5 min. Subsequently, the dehydrated slices were embedded in Epon812. Staining of the slices with 3% uranyl acetate (YS25690U, YaJi Biological, China) and lead citrate (L885990, MACKLIN, China) was performed at room temperature for 15 min. Finally, the slices were observed and photographed under a transmission electron microscope (TEM, JEM‐1400PLUS, Japan) [39].

### 2.23 Nissl Staining

Fresh tissue was fixed in 10% neutral formalin for 48 h, then dehydrated and embedded. Sections (6–8 μm thick) were stained with cresyl violet in an incubator at 50°C–60°C for 25–50 min. After differentiation in ethanol, dehydration, and clearing in xylene, the sections were mounted. Nissl‐positive cells were counted from eight sections per group, selecting five random fields for cell counting [39].

### 2.24 Statistical Software and Data Analysis Methods

The study was conducted using R software version 4.2.1, compiled with the integrated development environment RStudio, also version 4.2.1. Perl language (version 5.30.0) was used for file processing, and Cytoscape version 3.7.2 was employed. Data analysis and graphing were performed using GraphPad Prism 8.0. The Shapiro–Wilk test was used to assess the normality of data distribution. For normally distributed data, measurement data were expressed as mean ± standard deviation (SD), and an independent sample *t*‐test was used to compare differences between the two groups. For multiple group comparisons, one‐way ANOVA was applied, followed by Tukey's post hoc test for pairwise comparisons. For comparing data across different time points, two‐way ANOVA was used, followed by Bonferroni post hoc tests. For data that did not follow a normal distribution, nonparametric tests such as the Mann–Whitney *U* test or Wilcoxon signed‐rank test were employed. Statistical significance was set at *p* < 0.05.

## 3 Results

### 3.1 Identification of Significantly Reduced Neuronal Cell Content in Brain Tissue of EP Patients Through scRNA‐Seq

**FIGURE 1:**
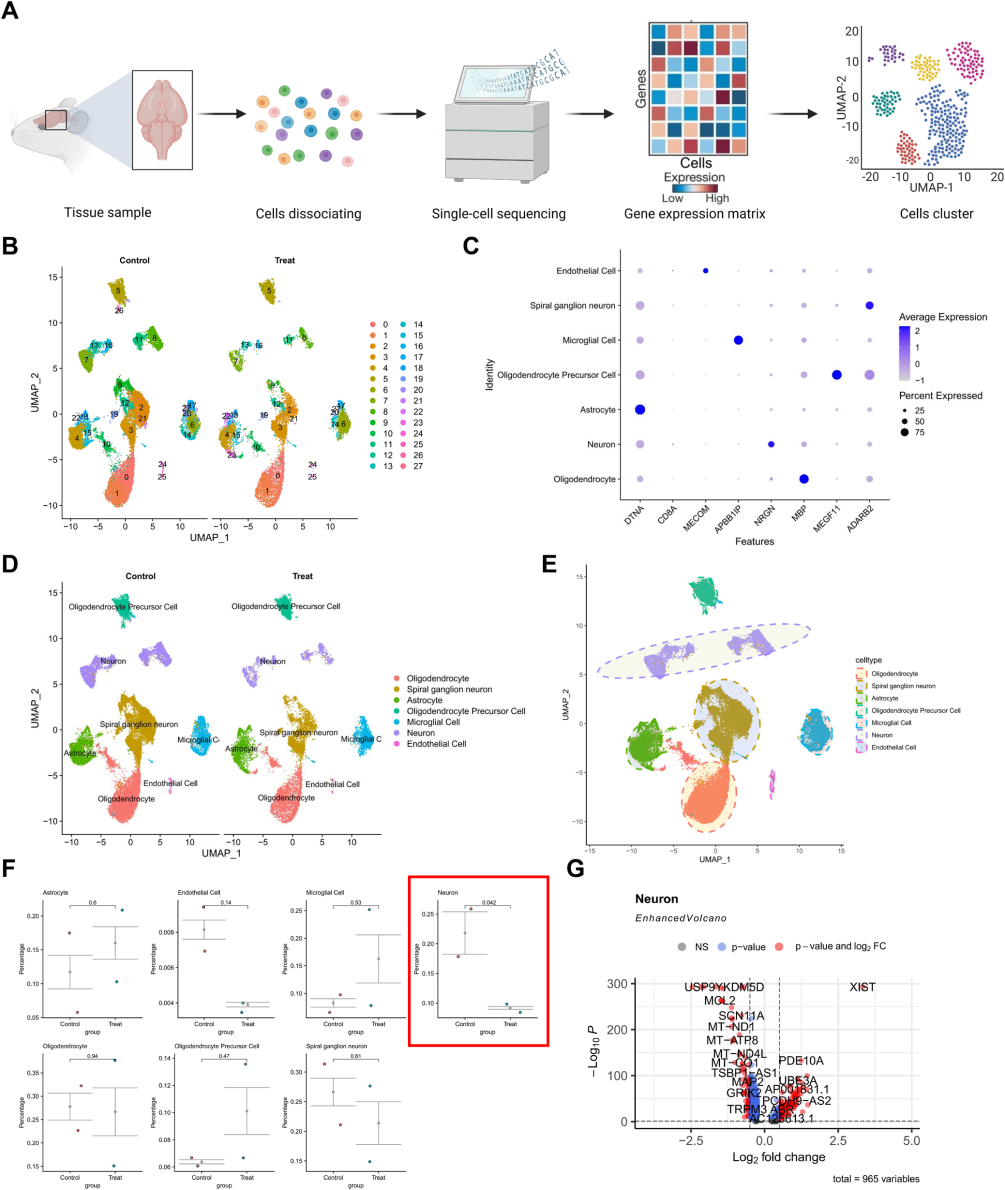
Cell clustering and annotation of scRNA‐Seq Data. (A) Schematic diagram of single‐cell data sample acquisition and analysis process; (B) Visualization of UMAP clustering results showing the cell aggregation and distribution in healthy samples (Control, *N* = 2) and EP samples (Treat, *N* = 2), with each color representing a cluster; (C) Expression of known lineage‐specific marker genes in different clusters of healthy samples (Control, *N* = 2) and EP samples (Treat, *N* = 2), where deeper blue indicates higher average expression levels, and larger circles represent more cells expressing the gene; (D) Visualization of cell annotation results in healthy group (*N* = 2) and EP group (*N* = 2) based on UMAP clustering, with each color representing a cell subtype; (E) Circular visualization of UMAP clustering results showing the cell aggregation and distribution in healthy and EP samples; (F) Significance levels of cell content differences between healthy group (*N* = 2) and EP group (*N* = 2) analyzed by T‐test, where the p‐values denote the significance of intergroup cell content differences; (G) Volcano plot of differentially expressed genes between Neuron cells in healthy group (*N* = 2) and EP group (*N* = 2) samples, where red dots on the left of the dashed line represent genes highly expressed in EP group samples, and dots on the right represent genes with low expression in EP group samples.

To investigate the neuronal cell content in EP patients, we analyzed scRNA‐seq data from two EP patients (T1‐2) and two healthy controls (C1‐2) from the GEO dataset GSE190452 (Figure 1A). Using the Seurat package, we integrated the sequencing data and assessed metrics such as the number of detected genes (nFeature_RNA), mRNA molecules (nCount_RNA), and the percentage of mitochondrial genes (percent.mt). Most cells exhibited nFeature_RNA < 5000, nCount_RNA < 20,000, and percent.mt < 20% (Figure S2A). After filtering low‐quality cells based on the criteria 200 < nFeature_RNA < 5000 and percent.mt < 25, we obtained an expression matrix with 5690 genes and 15,759 cells. Correlation analysis showed r = −0.13 between nCount_RNA and percent.mt, *r* = 0.86 between nCount_RNA and nFeature_RNA, confirming good data quality (Figure S2B).

Highly variable genes were screened for downstream analysis, and the top 2000 genes were selected based on expression variance (Figure S2C). Cell cycle scoring was computed using the CellCycleScoring function to determine the cell cycle of the samples (Figure S2D), followed by initial data normalization. Subsequently, based on the selected highly variable genes, PCA was employed to linearly reduce the data dimensions, yielding a PCA plot (Figure S2E). Here, we depict the heat map of the major correlating gene expressions for PC_1–PC_6 (Figure S2F). Batch correction using the Harmony package was performed to mitigate batch effects in the sample data, enhancing the accuracy of cell clustering (Figure S2G,H), along with utilizing ElbowPlot to sort the PCs by SD (Figure S2I). The results indicate that PC_1–PC_20 effectively capture the information contained in the selected highly variable genes, signifying substantial analytical importance.

Subsequently, the UMAP algorithm was utilized to perform nonlinear dimension reduction on the top 30 PCs, with a resolution set to 1 for cluster analysis (Figure S3). The cluster analysis revealed 28 clusters, showcasing the expression profile of marker genes for each cluster (Figure 1B). By researching relevant literature for cell lineage‐specific marker genes and utilizing the online resource CellMarker for cell annotation (Figure 1C), a total of seven cell types were identified: Astrocyte, endothelial cell, microglial cell, neuron, oligodendrocyte, oligodendrocyte precursor cell, and spiral ganglion neuron (Figure 1D,E). The *T*‐test results highlighted that neuron exhibited the most significant content difference in brain tissues between healthy individuals and EP patients (Figure 1F). Subsequently, a differential analysis of gene expression in neurons from brain tissues of healthy individuals and EP patients identified 480 genes with high expression levels in the EP group and 485 genes with low expression levels (Figure 1G).

In summary, the results indicate that brain tissue samples from EP patients can be categorized into 28 clusters comprising seven distinct cell types, with neuron exhibiting the most significant content difference in brain tissues between healthy individuals and EP patients.

### 3.2 MCL1 as a Significant Regulatory Factor in the Pathogenesis of EP

**FIGURE 2:**
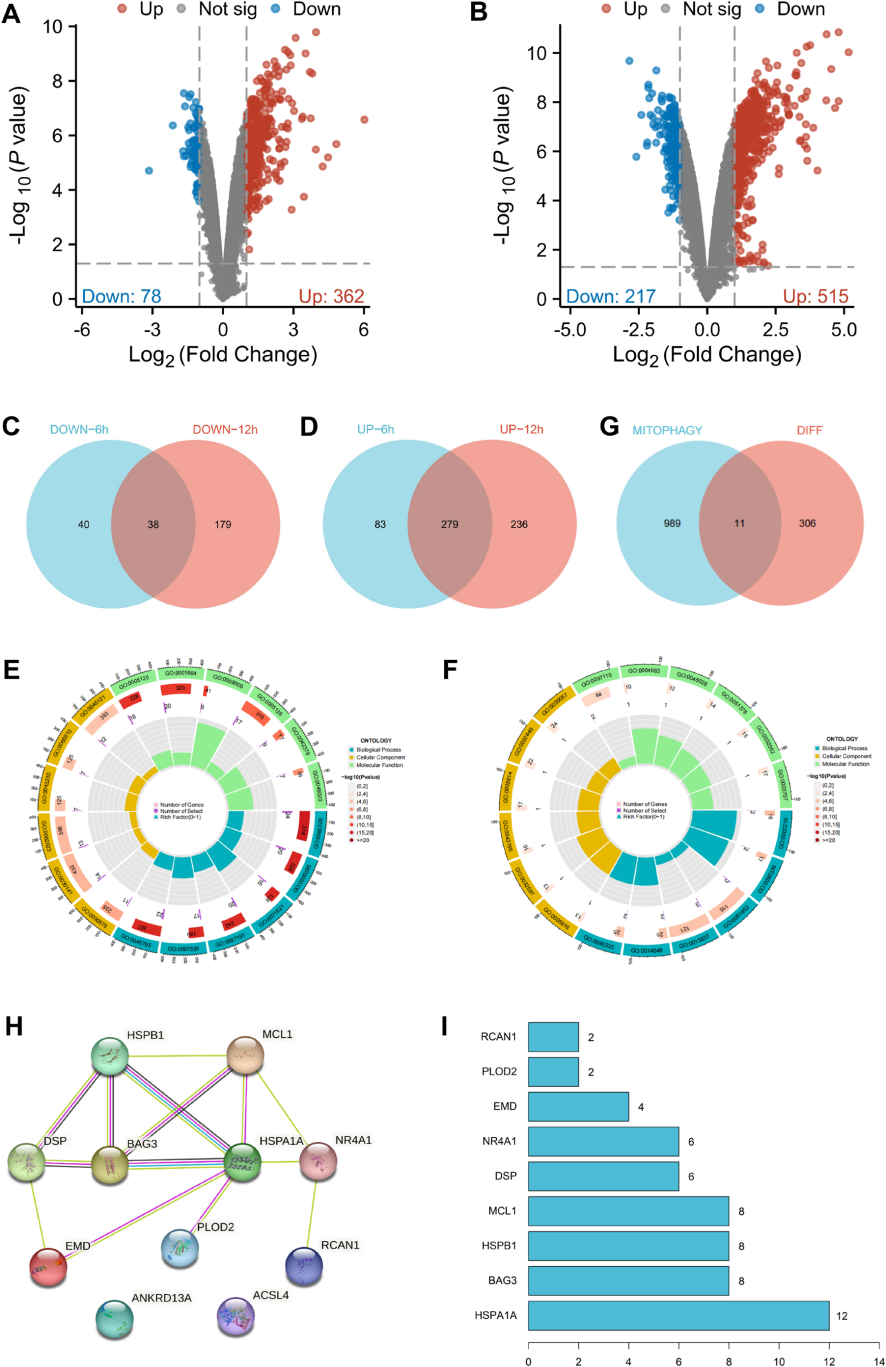
Identification of key regulators of mitophagy in the pathogenesis of EP. (A) Volcano plot showing differential gene expression in the hippocampus of mice at 6 h post‐injection, with blue dots indicating downregulated genes, red dots indicating upregulated genes, and gray dots indicating no significant difference, *n* = 3; (B) Volcano plot showing differential gene expression in the hippocampus of mice at 12 h post‐injection, with color coding as in (A); (C) Venn diagram depicting the intersection of downregulated genes in the hippocampus of mice at 6 and 12 h; (D) Venn diagram depicting the intersection of upregulated genes in the hippocampus of mice at 6 and 12 h; (E) Enrichment analysis results of intersecting upregulated genes, with color representation of GO categories (BP in blue, CC in green, MF in red, darker colors indicating more significant enrichment); (F) Enrichment analysis results of intersecting downregulated genes, following the same color scheme as in (E); (G) Venn diagram showing the intersection of differentially expressed genes with genes related to mitophagy retrieved from the GeneCard online database; (H) PPI network graph of proteins encoded by significantly differentially expressed genes related to mitophagy; (I) Bar graph depicting the top 10 genes ranked by node count in the PPI network of significantly differentially expressed genes related to mitophagy.

To gain a deeper understanding of the molecular and cellular mechanisms of EP, we established a mouse model of EP by microinjection of sodium valproate into the hippocampus and collected hippocampal tissues at 6 and 12 h post‐injection for high‐throughput transcriptome sequencing. Analysis of the transcriptomic data revealed 440 genes with significant differential expression at 6 h post‐injection (Figure 2A) and 732 genes at 12 h post‐injection (Figure 2B). Intersection analysis of significantly upregulated and downregulated genes yielded 279 significantly upregulated genes and 38 significantly downregulated genes (Figure 2C,D).

Gene Ontology enrichment analysis indicated that upregulated genes were involved in processes such as apoptotic signaling and cell chemotaxis, while downregulated genes were linked to neurotransmitter metabolism and external stimulus responses (Figure 2E,F). The enrichment results of differentially expressed genes revealed the activation of apoptotic pathways and the inhibition of neurotransmitter‐related metabolic pathways in the pathogenesis of EP.

The death of neuronal cells is commonly considered a pathological consequence of brain injuries leading to EP and is believed to be a causative factor in the onset of EP. Excitotoxic necrosis and apoptosis‐related pathways are the primary mechanisms underlying neuronal cell death [57]. Among these, mitophagy plays a crucial role in the process of cell apoptosis and excitotoxicity [10].

From the GeneCard database, we identified 11 mitophagy‐related genes with differential expression during EP (Figure 2G). Additionally, a PPI network was constructed to analyze the interactions between these proteins, and the number of connections between genes was calculated to assess their core importance. The analysis identified four genes as core nodes: HSPA1A, BAG3, HSPB1, and MCL1 (Figure 2H,I). Among these, HSPA1A, BAG3, and HSPB1 showed significantly higher expression in EP samples, while MCL1 exhibited significantly lower expression (Figure S4A,B). MCL1 has the ability to switch between autophagy and apoptosis functions under conditions of energy stress in a developmental regulatory manner, and literature reports suggest it may be an important target for neurological diseases [58]. Consequently, we chose the core factor MCL1 to further explore its regulatory function in the pathogenesis of EP.

### 3.3 Protective Role of MCL1 in Mouse EP

**FIGURE 3:**
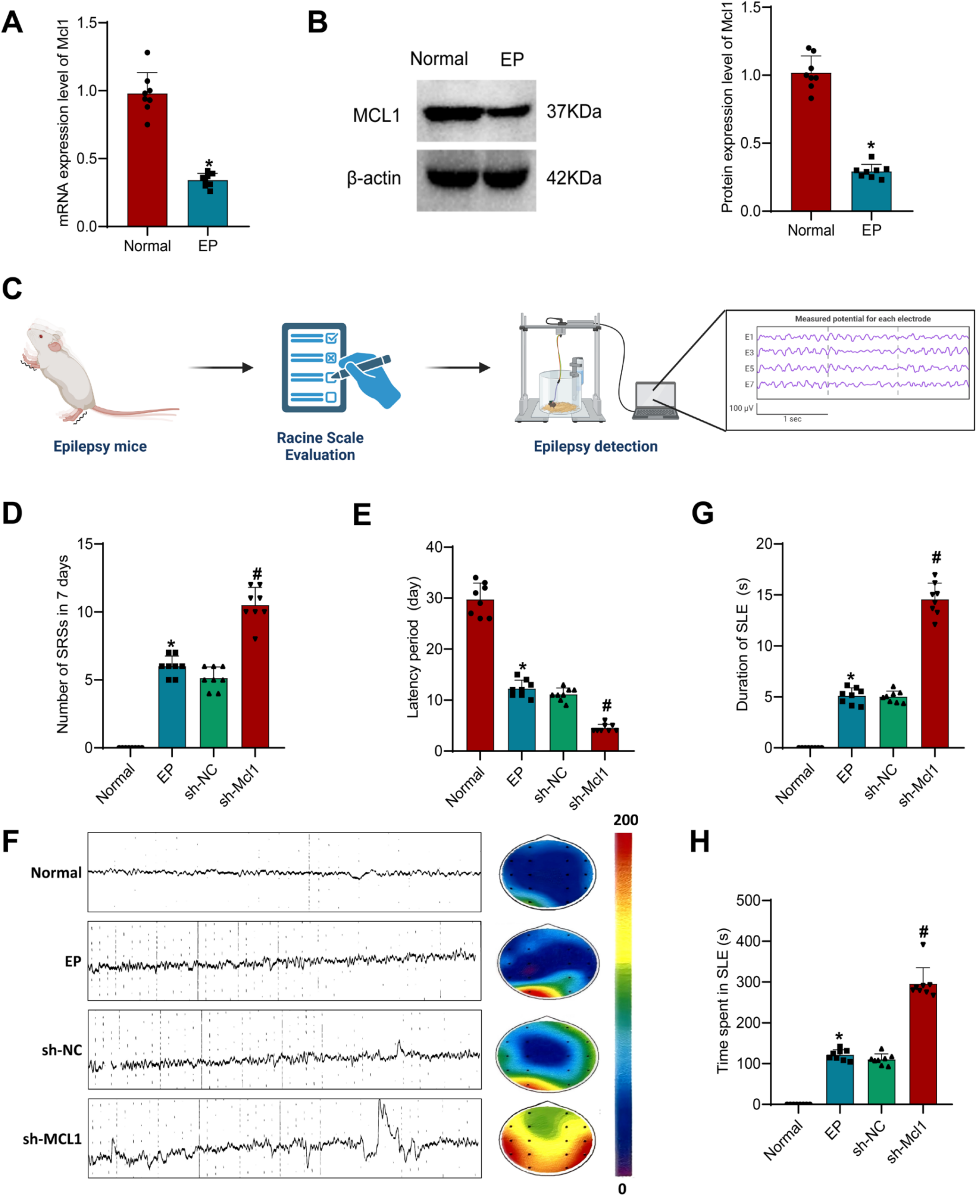
The expression of MCL1 and its impact on the onset of EP. (A) RT‐qPCR analysis of Mcl1 mRNA expression in the hippocampal tissues of normal mice and mice with EP; (B) Western Blot experiment investigating the protein expression of Mcl1 in the hippocampal tissues of normal mice and mice with EP; (C) Flowchart of the EP detection process in mice; (D) Spontaneous seizure frequency visible to the naked eye under behavioral monitoring, graded using the Racine scale; (E) Latency period of EP occurrence; (F) Local field potential spectra of mice in each group, with the heatmap on the right showing the frequency changes in brain electrical activity over time, where red indicates high‐intensity electrical activity; (G) Duration of EP in each group of mice; (H) Total duration of EP in each group of mice. * indicates a significance level of *p* < 0.05 compared to the Normal group; # indicates a significance level of *p* < 0.05 compared to the sh‐NC group. Mouse experiments, *n* = 8.

To investigate the expression of MCL1 in the occurrence of EP in mice, we collected mouse brain tissues and performed RT‐qPCR and Western blot experiments to detect its expression in the brain tissues. The results showed that compared to the normal group, the mRNA and protein expression of MCL1 in the brain tissues of the EP group of mice were significantly decreased (Figure 3A,B).

In order to explore the impact of MCL1 on EP seizures, we utilized lentiviral silencing of MCL1 expression (Figure S5A,B) and selected the sequence with the optimal silencing efficiency for subsequent experiments. We constructed an EP model through microinjection and assessed mouse behavior (Figure 3C). The findings indicated a significant increase in seizure frequency and a decrease in latency in the EP group compared to the normal group. Moreover, the sh‐MCL1 group exhibited a further significant increase in seizure frequency and a significant reduction in latency compared to the sh‐NC group (Figure 3D,E). Additionally, LFP was recorded in mice, revealing obvious EP‐like discharges in all groups except the normal group. The EP group displayed pronounced EP‐like pattern discharges, with SLE notably occurring. Furthermore, the total duration of discharges was significantly increased in the sh‐MCL1 group compared to the sh‐NC group (Figure 3F–H).

In conclusion, these results indicate that MCL1 plays a protective role in EP seizures in mice.

### 3.4 Involvement of MCL1 in Mitophagy‐Mediated Neuronal Damage

Mitochondria are the main site of ROS generation, and mitochondrial dysfunction is a common pathological feature of neurological diseases, particularly in the onset of EP. This is primarily due to elevated levels of mitochondrial ROS and decreased intracellular ATP levels resulting from oxidative stress, which promote the production and aggregation of pathogenic proteins [59], ultimately leading to neuronal damage during epileptic seizures [60]. To maintain normal cellular physiological processes, mitophagy acts to remove damaged mitochondria, initiating cell protective mechanisms, thereby playing a crucial role in delaying the progression of neurological disorders [61].

**FIGURE 4:**
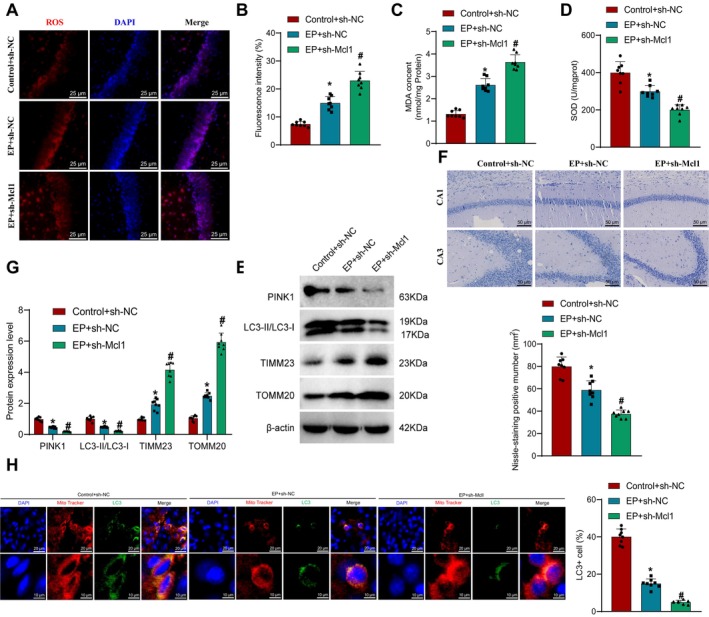
Impact of MCL1 on neuronal injury. (A) Evaluation of ROS levels in the hippocampal tissue of mice in each group using the DCFH‐DA probe method. ROS is represented in red, while DAPI is represented in blue. Scale bar = 25 μm; (B) Quantitative analysis of ROS detection fluorescence. Five independent fields were assessed per group, *n* = 5; (C) Measurement of MDA content in the hippocampal tissue using a commercial assay kit; (D) Detection of SOD levels in the hippocampal tissue of mice using a commercial assay kit; (E) Evaluation of the expression of key mitophagy proteins (PINK1, LC3‐I, LC3‐II, TIMM23, TOMM20) in the hippocampal tissue of mice through Western Blot; (F) Assessment of neuronal status and Nissl body count using Nissl staining method. Scale bar = 50 μm; (G) Statistical analysis of Nissl body counts observed under a microscope. Five independent fields were counted per group, *n* = 5; (H) Co‐localization of mitochondria and LC3 in neuronal cells of each group observed by confocal laser microscopy (scale bar: 25 μm). * indicates significant difference compared to the Control + sh‐NC group, *p* < 0.05, # indicates significant difference compared to the EP + sh‐NC group, *p* < 0.05. *n* = 8 for mouse experiments.

Subsequently, we investigated the impact of MCL1 on oxidative stress, mitophagy, and neuronal damage. Using the DCFH‐DA probe method, we detected the accumulation of ROS in the mouse hippocampus and measured the levels of MDA and SOD in the hippocampal tissues using assay kits. The results revealed that compared to the Control + sh‐NC group, the EP + sh‐NC group exhibited significantly higher accumulation of ROS and MDA in the mouse hippocampal tissues, along with a marked decrease in SOD levels. Furthermore, compared to the EP + sh‐NC group, the EP + sh‐Mcl1 group displayed a significantly increased accumulation of ROS and MDA in the mouse hippocampal tissues, accompanied by a notable reduction in SOD levels (Figure 4A–D). In instances of impaired mitochondrial function, PINK1 accumulates on the outer membrane of mitochondria and cooperates with Parkin to initiate the process of mitophagy. During this process, LC3‐I is converted to the LC3‐II form that binds to the mitochondrial membrane, facilitating the engulfment and degradation of damaged mitochondria within autophagic vacuoles. In normal mitochondrial function, TOMM20 and TIMM23, as membrane proteins of mitochondria, assist in the transport of cytoplasmic proteins containing mitochondrial localization signals into the inner compartments of the mitochondria [10]. Additionally, we conducted Western blot experiments to examine key proteins involved in mitophagy (PINK1, LC3‐I, LC3‐II, TIMM23, TOMM20). The analysis showed that compared to the Control + sh‐NC group, the EP + sh‐NC group exhibited a significant decrease in PINK1 protein expression, a reduction in the ratio of LC3‐II/LC3‐I protein expression, and a notable increase in TIMM23 and TOMM20 protein expression. Furthermore, compared to the EP + sh‐NC group, the sh‐Mcl1 group demonstrated a further reduction in PINK1, a significant increase in TIMM23 and TOMM20 protein expression, and a marked decrease in the ratio of LC3‐II/LC3‐I protein expression (Figure 4E).

Subsequently, we evaluated the morphology and viability of neurons through Nissl staining. The results revealed that compared to the Control + sh‐NC group, neurons in the EP + sh‐NC group in the hippocampus of mice showed incomplete and ruptured cells. Following MCL1 knockdown, the neuronal damage was exacerbated (Figure 4F,G). Confocal laser microscopy was utilized to assess the colocalization of mitochondria and LC3 to monitor changes in mitophagy. As depicted in Figure 4H, colocalization of mitochondria and LC3 in neuronal cells decreased in the EP + sh‐NC group relative to the Control + sh‐NC group. Furthermore, compared to the EP + sh‐NC group, this colocalization further decreased after the MCL1 knockdown.

These findings suggest that MCL1 knockdown can exacerbate neuronal damage by reducing mitophagy.

### 3.5 LITAF Promotes MCL1 Ub to Inhibit Mitophagy

**FIGURE 5:**
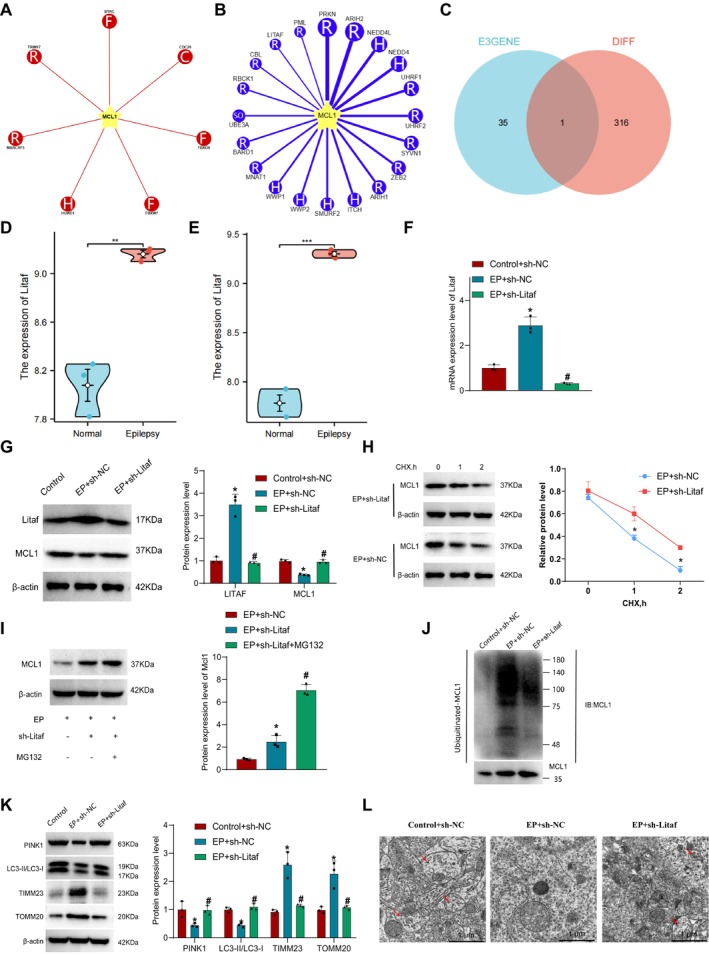
The impact of LITAF on MCL Ub and mitophagy. (A) Seven E3 ubiquitin ligases confirmed to regulate MCL1 Ub obtained from the Ubibrowser database; (B) Twenty‐nine E3 ubiquitin ligases potentially regulating MCL1 Ub obtained from the Ubibrowser database; (C) Venn diagram showing the intersection of identified E3 ubiquitin ligases regulating MCL1 with differentially expressed genes; (D) Expression of Litaf in the mouse hippocampus at 6 h post‐injection; (E) Expression of Litaf in the mouse hippocampus at 12 h post‐injection, *n* = 3; (F) RT‐qPCR analysis of Litaf mRNA expression in isolated neuronal cells; (G) Western Blot analysis of Litaf and MCL1 protein expression in isolated neuronal cells; (H) Protein imprinting assay to detect MCL1 protein levels in neuronal cells treated with the protein synthesis inhibitor CHX; (I) Examination of MCL1 protein expression after treatment with the proteasome inhibitor MG132 using protein imprinting; (J) Immunoblot analysis of MCL1 Ub levels in neuronal cells; (K) Western Blot analysis of key proteins involved in mitophagy in isolated neuronal cells; (L) Transmission electron microscopy observation of the morphology of neurons and mitochondria in the hippocampal tissue, scale bar = 1μm. * indicates *p* < 0.05 compared to the Control+sh‐NC group, ** represents *p* < 0.01 compared to the Normal group; *** signifies *p* < 0.001 compared to the Normal group, # indicates *p* < 0.05 compared to the EP + sh‐NC group. All cell experiments were repeated three times.

MCL1 is an unstable protein whose stability is regulated by the ubiquitin–proteasome system (UPS). Specific E3 ubiquitin ligases can mediate the Ub of MCL1, leading to its degradation and altering its regulation of mitophagy [62]. To investigate the regulatory mechanism of MCL1 Ub on mitophagy in the pathogenesis of EP, we first predicted upstream ubiquitin ligases of MCL1 through the Ubibrowser database. The results revealed 36 E3 ubiquitin ligases interacting with MCL1, including seven known E3 ligases that regulate MCL1 Ub, as well as 29 E3 ligases predicted to interact with a confidence score between 0.825 and 0.896 (Figure 5A,B).

Furthermore, we identified intersecting genes between E3 ubiquitin ligases interacting with MCL1 and differentially expressed genes in EP, leading to the identification of the intersecting gene, LITAF (Figure 5C). Results from RT‐qPCR experiments showed significantly higher expression of the predicted E3 ubiquitin ligase LITAF during the onset of EP (Figure 5D,E). Subsequently, we predicted the sites affected by LITAF on MCL1 Ub and the recognition sequence of substrates through the Ubibrowser database, revealing nine sites where LITAF affects MCL1 Ub and a potential E3 recognition motif (Figure S6).

To investigate the regulatory role of LITAF in EP seizures, we isolated neurons from normal and epileptic mice for in‐depth exploration (Figure S7A,B). Using lentiviral transfection, we silenced LITAF in neurons (Figure S7C,D) and examined Litaf expression through RT‐qPCR and Western blot experiments. The results indicated that compared to the Control + sh‐NC group, the EP + sh‐NC group showed a significant increase in mRNA and protein expression of LITAF, while the protein expression level of MCL1 decreased notably. In contrast, compared to the EP + sh‐NC group, the EP + sh‐Litaf group exhibited a significant decrease in both mRNA and protein levels of LITAF, along with a notable increase in MCL1 protein expression (Figure 5F,G), suggesting high expression of LITAF in EP samples.

To investigate whether LITAF regulates mitophagy by ubiquitinating MCL1 during EP onset, we examined the Ub levels of MCL1 in silenced neurons through Western blot. The results of protein stability assays indicated that after treatment with the protein synthesis inhibitor cycloheximide (CHX), the EP + sh‐Litaf group showed a faster decrease in MCL1 protein levels compared to the EP + sh‐NC group (Figure 5H), indicating that silencing LITAF increased the stability of MCL1 and reduced its degradation. Subsequently, we treated neurons with the proteasome inhibitor MG132 (10 μM) and found that LITAF promoted the degradation of MCL1 (Figure 5I). The results showed that compared to the Control + sh‐NC group, the EP + sh‐NC group exhibited a significant increase in MCL1 Ub levels, while the EP + sh‐Litaf group showed a significant decrease in MCL1 Ub levels (Figure 5J). Furthermore, we observed mitophagy in neurons using electron microscopy and detected the expression of proteins related to mitophagy through Western blot. The findings revealed that compared to the Control+sh‐NC group, the EP + sh‐NC group displayed a significant reduction in the ratio of PINK1, LC3‐II/LC3‐I protein expression, increased expression of TIMM23 and TOMM20 proteins, and a decrease in double‐membrane autophagosome quantity. In contrast, the EP + sh‐Litaf group showed an increase in the ratio of LC3‐II/LC3‐I protein expression, a decrease in TIMM23 and TOMM20 protein expression, an increase in PINK1 protein expression, and an augmentation in double‐membrane autophagosome quantity (Figure 5K,L).

Overall, these results indicate that during the onset of EP, LITAF can promote MCL1 Ub and inhibit mitophagy.

### 3.6 LITAF Promotes MCL1 Ub to Inhibit Mitophagy and Induce Neuronal Cell Apoptosis

**FIGURE 6:**
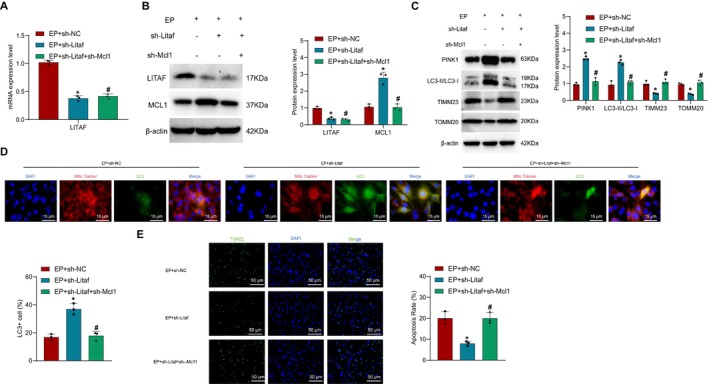
Impact of LITAF regulation on MCL Ub on mitophagy and neuronal cell Apoptosis. (A) RT‐qPCR experiments to measure the expression levels of LITAF in neuronal cells of each group; (B) Western blot experiments to examine the expression levels of LITAF and MCL1 in neuronal cells of each group; (C) Western blot experiments to assess the expression of key proteins related to mitophagy in isolated neuronal cells; (D) Co‐localization results of mitochondria and LC3 in neuronal cells of each group observed by confocal laser scanning microscopy (scale bar: 15 μm); (E) TUNEL assay to determine the apoptosis rate of neuronal cells in each group (Scale bar = 50 μm). * indicates significant difference compared to the EP + sh‐NC group, *p* < 0.05, # indicates significant difference compared to the EP + sh‐Litaf group, *p* < 0.05. The cell experiments were repeated three times.

To further investigate the impact of LITAF and MCL1 on neuronal cell mitophagy and cell damage, we concurrently knocked down LITAF and MCL1 in neuronal cells. Experimental results confirmed that compared to the EP + sh‐NC group, the EP + sh‐Litaf group exhibited a significant decrease in LITAF expression and a marked increase in MCL1 protein levels. Moreover, in comparison to the EP + sh‐Litaf group, the EP + sh‐Litaf+sh‐Mcl1 group showed stable LITAF expression but a significant reduction in MCL1 protein levels (Figure 6A,B). Additionally, compared to the EP + sh‐NC group, the EP + sh‐Litaf group showed a notable increase in the expression ratio of PINK1 and LC3‐II/LC3‐I proteins, along with a decrease in TIMM23 and TOMM20 protein levels. Furthermore, when compared to the EP + sh‐Litaf group, the EP + sh‐Litaf + sh‐Mcl1 group displayed a reduced expression ratio of PINK1 and LC3‐II/LC3‐I proteins, accompanied by a significant increase in TIMM23 and TOMM20 protein levels (Figure 6C).

Confocal laser microscopy observations revealed that the co‐localization in the EP + sh‐Litaf group was higher than that in the EP + sh‐NC group, while the EP + sh‐Litaf + sh‐Mcl1 group exhibited a significant reduction in co‐localization compared to the EP + sh‐Litaf group (Figure 6D). TUNEL staining results indicated a significant decrease in apoptosis of neuronal cells in the EP + sh‐Litaf group compared to the EP + sh‐NC group, whereas the EP + sh‐Litaf + sh‐Mcl1 group showed a significant increase in neuronal cell apoptosis compared to the EP + sh‐Litaf group (Figure 6E).

These findings suggest that during the onset of EP, LITAF can promote MCL1 Ub to inhibit mitophagy and induce neuronal cell apoptosis.

### 3.7 LITAF Inhibits Mitophagy and Promotes Epileptogenesis by Facilitating MCL‐1 Ub

**FIGURE 7:**
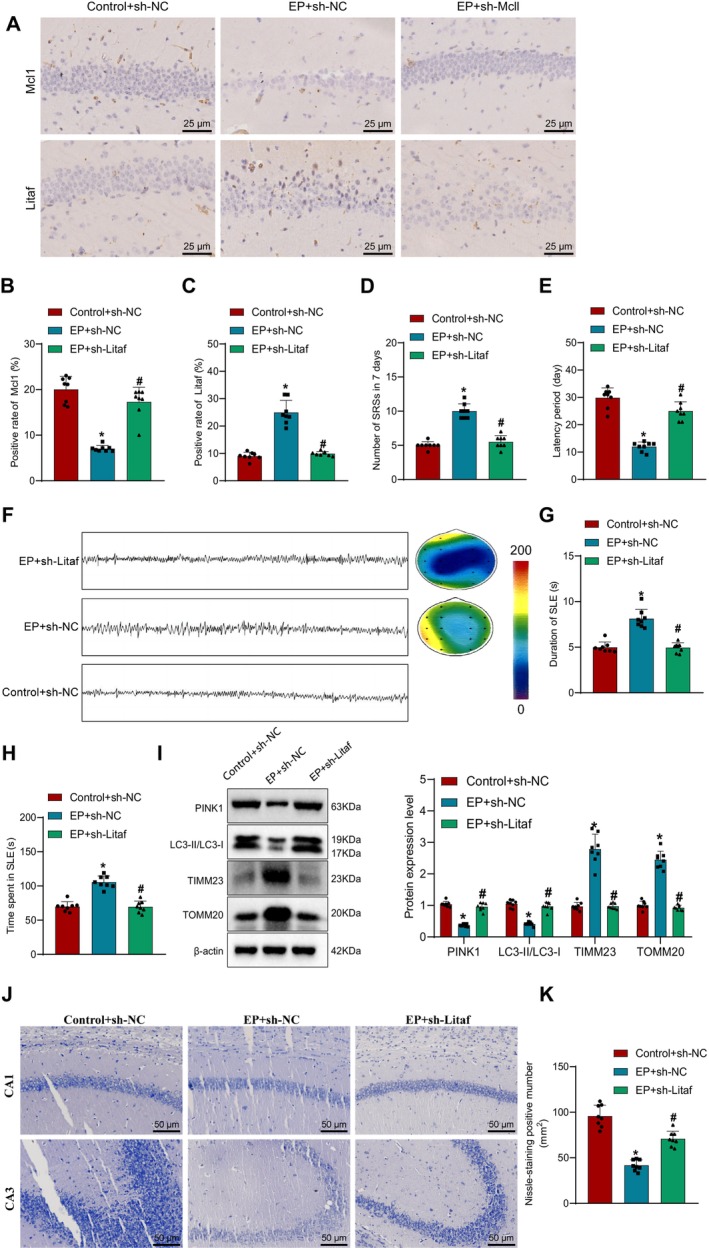
Investigation of LITAF promoting MCL‐1 Ub and activating mitophagy in influencing epileptic seizures. (A) Immunohistochemistry detection of protein expression of Mcl1 and Litaf in mouse hippocampal tissue, scale bar = 25 μm; (B) Statistical chart of positive protein expression of Mcl1; (C) Statistical chart of positive protein expression of Litaf; (D) Spontaneous EP seizure frequency visible to the naked eye under behavioral monitoring, grading can be done using the Racine scale; (E) Latency period of EP occurrence; (F) Local field potential spectra in various groups of mice; (G) Duration of EP in different groups of mice; (H) Total duration of EP in different groups of mice; (I) Expression of key proteins of mitophagy in neuron cells separated by Western Blot experiment; (J) Assessment of neuron status and Nissl body quantity using Nissl staining method; (K) Statistical chart of Nissl body quantity observed under a microscope. * indicates *p* < 0.05 compared to the Control+sh‐NC group; # indicates *p* < 0.05 compared to the EP + sh‐NC group. Experimental sample size *n* = 8 mice.

To investigate the impact of LITAF on epileptogenesis, we utilized lentiviral transfection to downregulate LITAF expression. Immunohistochemistry was conducted to assess the protein expression of Mcl1 and Litaf in the hippocampal tissue of mice. The results revealed a significant increase in the expression of Mcl1 and Litaf in the EP + sh‐NC group compared to the Control + sh‐NC group. Furthermore, in comparison to the EP + sh‐NC group, the EP + sh‐Litaf group exhibited a notable elevation in Mcl1 expression while showing a significant decrease in Litaf expression (Figure 7A–C). Subsequently, behavioral assessments of the mice demonstrated that the frequency of EP seizures increased, and the latency period decreased in the EP + sh‐NC group compared to the Control + sh‐NC group. Conversely, in comparison to the EP + sh‐NC group, the EP + sh‐Litaf group displayed a marked reduction in EP seizure frequency and a significant increase in the latency period (Figure 7D,E). Additionally, local field potential recordings in mice indicated a significant increase in EP‐like discharge duration in the EP + sh‐NC group compared to the Control + sh‐NC group. Conversely, the total duration decreased significantly in the EP + sh‐Litaf group compared to the EP + sh‐NC group (Figure 7F–H).

Subsequent analysis involved the collection of hippocampal tissue from mice to evaluate the expression of mitophagy‐related proteins. The results demonstrated a significant decrease in PINK1 protein expression and a reduced LC3‐II/LC3‐I protein expression ratio in the EP + sh‐NC group compared to the Control + sh‐NC group. Additionally, the protein expression of TIMM23 and TOMM20 significantly increased. In contrast, compared to the EP + sh‐NC group, the EP + sh‐Litaf group exhibited an increased LC3‐II/LC3‐I protein expression ratio, a marked decrease in TIMM23 and TOMM20 protein expressions, and a significant increase in PINK1 protein expression (Figure 7I).

Furthermore, Nissl staining was utilized to investigate neuronal cell damage in the hippocampal tissue. The results indicated increased neuronal damage in the EP + sh‐NC group compared to the Control + sh‐NC group. Conversely, in comparison to the EP + sh‐NC group, neuronal cell damage was ameliorated in the EP + sh‐Litaf group (Figure 7J,K).

In conclusion, the findings suggest that LITAF can inhibit mitophagy and promote epileptogenesis by facilitating MCL‐1 Ub.

## 4 Discussion

Previous research has established EP as a neurological disorder marked by abnormal synchronization of neuronal activity [63]. This study further elucidates the role of mitophagy in the pathogenesis of EP [10]. Mitophagy, a cellular process that clears damaged mitochondria, is crucial for maintaining cell health and function [8, 9, 64]. While previous studies have linked mitochondrial dysfunction to various neurological disorders, the precise connection between EP and mitophagy has not been fully explored [65, 66].

In this study, we identified MCL1 as a core factor in EP pathogenesis through PPI network analysis. While MCL1 has traditionally been associated with the regulation of apoptosis, our research reveals a new role for MCL1 in preventing neuronal damage and mitigating EP [67]. MCL1 reduces neuronal damage by engaging in the mitophagy pathway, offering a novel therapeutic avenue. Specifically, MCL1 helps preserve mitochondrial integrity by inhibiting proapoptotic factors such as BAX and BAK. Reduced MCL1 expression can increase mitochondrial outer membrane permeability, activating mitophagy to remove damaged mitochondria and prevent excessive apoptosis [68].

LPS‐induced TNF‐alpha factor, though not previously associated with EP [69], was found to be significantly elevated during EP progression in this study. Our findings indicate that LITAF inhibits mitophagy by promoting the ubiquitination (Ub) of MCL1, providing new biological insights into LITAF's role and presenting it as a potential therapeutic target for EP.

In the pathogenesis of EP, it has been observed that inhibiting mitophagy exacerbates neuronal oxidative stress and promotes neuronal damage. Oxidative stress has long been associated with the onset and progression of many neurodegenerative diseases [70, 71, 72]. This study further underscores the critical role of oxidative stress in EP and suggests that mitophagy may serve as a new pathway for regulating oxidative stress.

Through high‐throughput transcriptome sequencing and GO enrichment analysis, we can rapidly identify differentially expressed genes and related BP in the pathogenesis of EP [73]. Bioinformatics approaches enable us to discover potential therapeutic targets within massive datasets and expedite the research process [74]. This indicates that bioinformatics methods play an increasingly important role in future studies of neurological disorders [75, 76, 77].

Based on the above experiments, we have tentatively concluded that in the development of EP, the high expression of the E3 ubiquitin ligase LITAF promotes the degradation of MCL1 by facilitating MCL1 Ub, thereby inhibiting the level of mitophagy, exacerbating neuronal oxidative stress, leading to neuronal damage, hindering the protective role of MCL1, and promoting the onset of EP. This study offers new insights into the molecular mechanisms of EP, elucidating the role of E3 ubiquitin ligase LITAF in inhibiting mitophagy by promoting MCL1 Ub, thereby exacerbating neuronal oxidative stress and promoting neuronal damage. These findings provide us with more precise targets and deepen our understanding of the pathophysiological mechanisms of EP.

An in‐depth investigation of the interaction between the E3 ubiquitin ligase LITAF and MCL1 has provided novel therapeutic strategies for EP. Theoretically, inhibiting LITAF or regulating the Ub process of MCL1 may bring new breakthroughs in EP treatment. This study extensively utilized bioinformatics tools such as the GeneCard database and Ubibrowser database, effectively combining in vitro and in vivo experiments to explore the etiology of EP, demonstrating the significant value of bioinformatics in modern neurologic disorder research [78].

In the in vivo experiments, we confirmed that MCL1 knockdown led to a significant increase in the total duration of seizure‐like discharges in epileptic model mice, while Litaf knockdown resulted in a significant decrease in the total duration of seizure‐like discharges in epileptic model mice. Combined with in vitro experiments, the molecular mechanism was found to be that LITAF promotes MCL1 ubiquitination, inhibiting mitophagy and promoting neuronal apoptosis, thereby inducing EP onset. Although the study utilized in vitro and in vivo models to assess the impact of LITAF on MCL1 Ub, it mainly relied on lentiviral transduction technology, which could elicit nonspecific cellular responses, potentially affecting result interpretation. Furthermore, this study predominantly depended on murine models for research, and discrepancies in physiology and genetics exist between mice and humans, necessitating further validation in human subjects. The scRNA‐seq data used in the study were sourced from public databases, and a random selection of samples from the dataset was analyzed, potentially introducing biases such as geographical and ethnic backgrounds, disease progression, and treatment histories, which may influence the representativeness and generalizability of the data. High‐throughput transcriptomic sequencing may overlook crucial genes with low expression levels that might play pivotal roles in disease development. While the inhibition of LITAF or regulation of MCL1 Ub is theoretically feasible, its long‐term therapeutic efficacy and safety have not been thoroughly researched.

Future validation of this mechanism in human or more human‐like models is hoped to lay a solid foundation for clinical applications. Building upon these findings, further investigations into the interaction mechanisms between LITAF and MCL1 can lead to the identification of more precise and specific therapeutic targets. Future studies could utilize gene knockout mouse models, which can more accurately simulate the long‐term effects of gene inactivation, reducing the variability potentially introduced by lentiviral transduction. Gathering and expanding a broader range of samples from diverse populations and different types of EP for bioinformatic analysis can evaluate the universality and translatability of the discoveries. Additionally, integrating more bioinformatics tools and big data technologies can precisely uncover key genes and pathways related to EP, providing more accurate strategies for disease prevention and treatment.

## Author Contributions

**Fuli Min** and **Zhaofei Dong:** conceived and designed the study. **Shuisheng Zhong** and **Ze Li:** performed the experiments. **Hong Wu** and **Sai Zhang:** conducted data analysis. **Linming Zhang** and **Tao Zeng:** supervised the project and contributed to manuscript preparation. **Fuli Min** and **Zhaofei Dong:** contributed equally to the work. All authors reviewed and approved the final manuscript.

## Ethics Statement

The experimental procedures and animal handling protocols were approved by the Institutional Animal Ethics Committee of The Eighth Affiliated Hospital, Sun Yat‐Sen University (approved number: SYSU‐IACUC‐2020‐B0178).

## Consent

The authors have nothing to report.

## Conflicts of Interest

The authors declare no conflicts of interest.

## Supporting information


**Figure S1.** Schematic illustration of mouse modeling.

**Figure S2.** Quality control and PCA dimensionality reduction of scRNA‐Seq data. (A) Violin plots showing the distribution of the number of genes per cell (nFeature_RNA), number of mRNA molecules (nCount_RNA), and the percentage of mitochondrial genes (percent.mt) in scRNA‐seq data (*N* = 4). (B) Scatter plots illustrating the correlation between filtered data of nCount_RNA and percent.mt, nCount_RNA and nFeature_RNA, and nCount_RNA and percent.HB (*N* = 4). (C) Variance analysis is used to select highly variable genes, with red indicating the top 2000 highly variable genes and black representing genes with low variability. The top 10 genes in the highly variable gene set are labeled (*N* = 4). (D) Cell cycle status of each cell in scRNA‐seq data, where S.Score represents the S phase and G2M. The score represents the G2M phase (*N* = 4). (E) PCA analysis depicts the distribution of cells in PC_1 and PC_2, with each point representing a cell (*N* = 4). (F) Heatmap showing the expression levels of the top 20 genes most correlated with PC_1–PC_6 in PCA, where yellow signifies upregulation and purple indicates downregulation (*N* = 4). (G) Batch correction process diagram of Harmony, with the x‐axis representing the number of iterations (*N* = 4). (H) Distribution of cells in PC_1 and PC_2 after batch correction by Harmony, with each point representing a cell. (I) Distribution of SD of PCs, where important PCs exhibit higher SD (N = 4).

**Figure S3.** UMAP clustering tree plot of scRNA‐Seq data.

**Figure S4.** Expression of core genes in normal and epileptic mice. (A) Expression of four core genes in the hippocampus of epileptic mice 6 h post‐injection based on transcriptomic data; (B) Expression of four core genes in the hippocampus of epileptic mice 12 h post‐injection based on transcriptomic data. *n* = 3.

**Figure S5.** Validation of Lentivirus Silencing Efficiency. (A) Detection of Mcl1 mRNA expression in cells transfected with lentivirus using RT‐qPCR; (B) Evaluation of Mcl1 protein expression in cells transfected with lentivirus through Western Blot experiments. * indicates significance compared to the sh‐NC group, *p* < 0.05. The cell experiments mentioned were repeated three times.

**Figure S6.** Sequence view of the LITAF‐MCL1 interaction. Ub sites are indicated by yellow highlights.

**Figure S7.** Identification and validation of primary neuronal cells with lentiviral silencing efficiency. (A) Immunofluorescence staining for purity assessment of isolated neuronal cells in the Normal group, where green represents neuronal cells and blue indicates DAPI, scale bar = 25 μm; (B) Immunofluorescence staining for purity assessment of isolated neuronal cells in the EP group, where green represents neuronal cells and blue indicates DAPI, scale bar = 25 μm; (C) RT‐qPCR detecting the mRNA expression of Litaf in cells post lentiviral transfection; (D) Western blot experiment detecting the protein expression of Litaf in cells post lentiviral transfection. * indicates *p* < 0.05 compared to the sh‐NC group. The cell experiments involved were repeated three times.

**Table S1.** Cell types and marker genes.

## Data Availability

The data that support the findings of this study are available from the corresponding author upon reasonable request.
