# Supplementary figures and images for: Impact of LITAF on Mitophagy and Neuronal Damage in Epilepsy via MCL‐1 Ubiquitination
Source: CNS Neurosci Ther. 2025 Jan 7;31(1):e70191. doi: 10.1111/cns.70191 (PMC11705406; doi:10.1111/cns.70191)

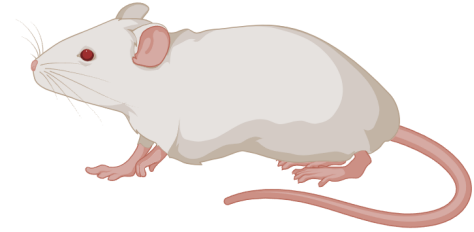

**Normal mouse**

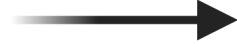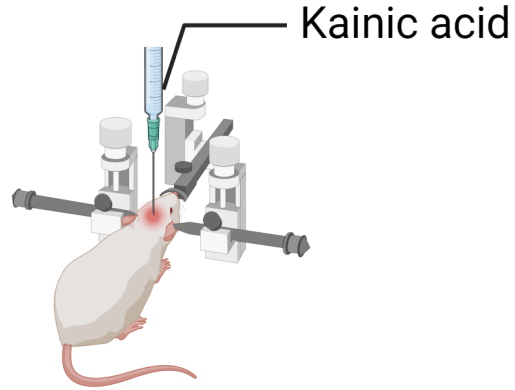

**Stereotaxic injection**

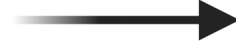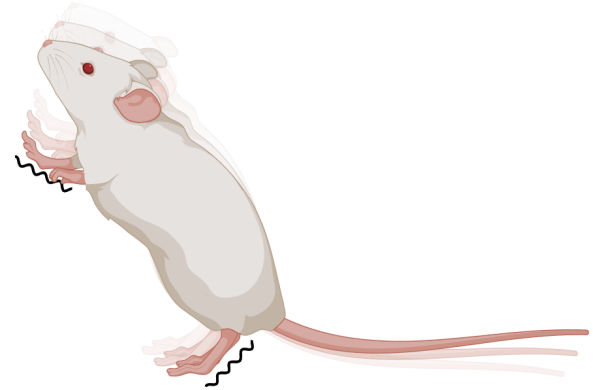

**Epilepsy mice**

Supplement: Supplementary file 1 — Figure S1. Schematic illustration of mouse modeling. [file CNS-31-e70191-s008.pdf]

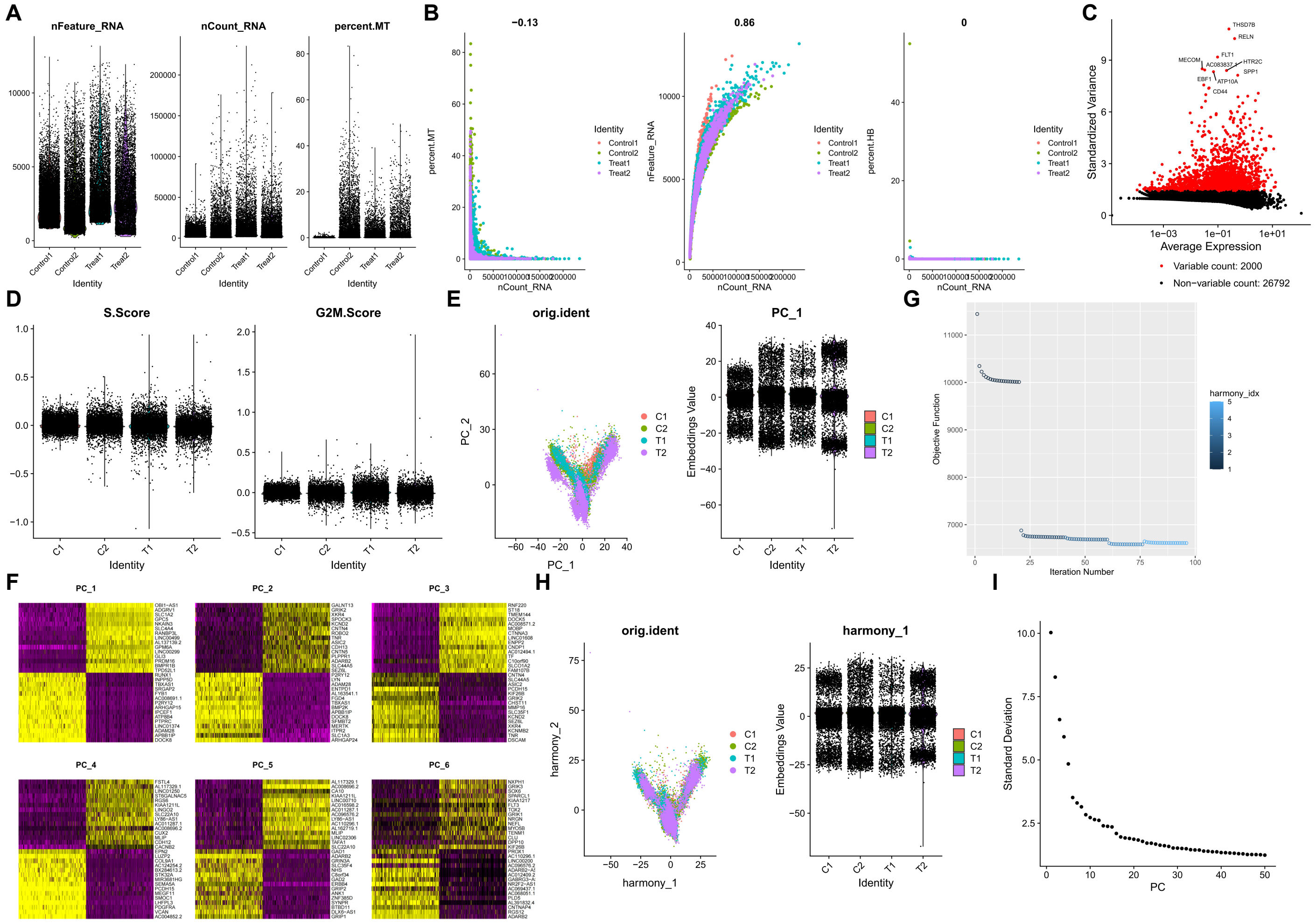

Supplement: Supplementary file 2 — Figure S2. Quality control and PCA dimensionality reduction of scRNA‐Seq data. (A) Violin plots showing the distribution of the number of genes per cell (nFeature_RNA), number of mRNA molecules (nCount_RNA), and the percentage of mitochondrial genes (percent.mt) in scRNA‐seq data (N = 4). (B) Scatter plots illustrating the correlation between filtered data of nCount_RNA and percent.mt, nCount_RNA and nFeature_RNA, and nCount_RNA and percent.HB (N = 4). (C) Variance analysis is used to select highly variable genes, with red indicating the top 2000 highly variable genes and black representing genes with low variability. The top 10 genes in the highly variable gene set are labeled (N = 4). (D) Cell cycle status of each cell in scRNA‐seq data, where S.Score represents the S phase and G2M. The score represents the G2M phase (N = 4). (E) PCA analysis depicts the distribution of cells in PC_1 and PC_2, with each point representing a cell (N = 4). (F) Heatmap showing the expression levels of the top 20 genes most correlated with PC_1–PC_6 in PCA, where yellow signifies upregulation and purple indicates downregulation (N = 4). (G) Batch correction process diagram of Harmony, with the x‐axis representing the number of iterations (N = 4). (H) Distribution of cells in PC_1 and PC_2 after batch correction by Harmony, with each point representing a cell. (I) Distribution of SD of PCs, where important PCs exhibit higher SD (N = 4). [file CNS-31-e70191-s004.pdf]

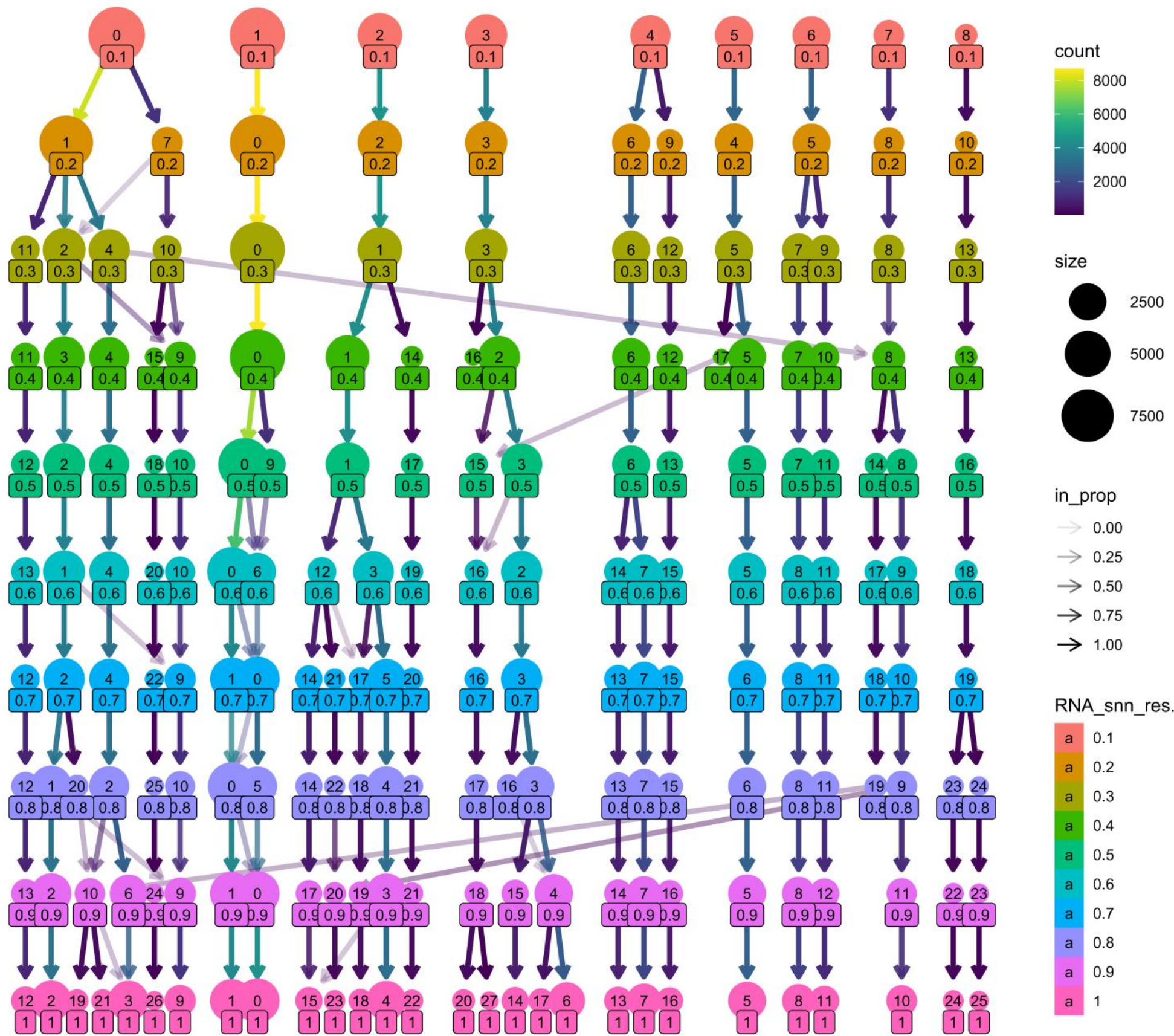

Supplement: Supplementary file 3 — Figure S3. UMAP clustering tree plot of scRNA‐Seq data. [file CNS-31-e70191-s005.pdf]

**A**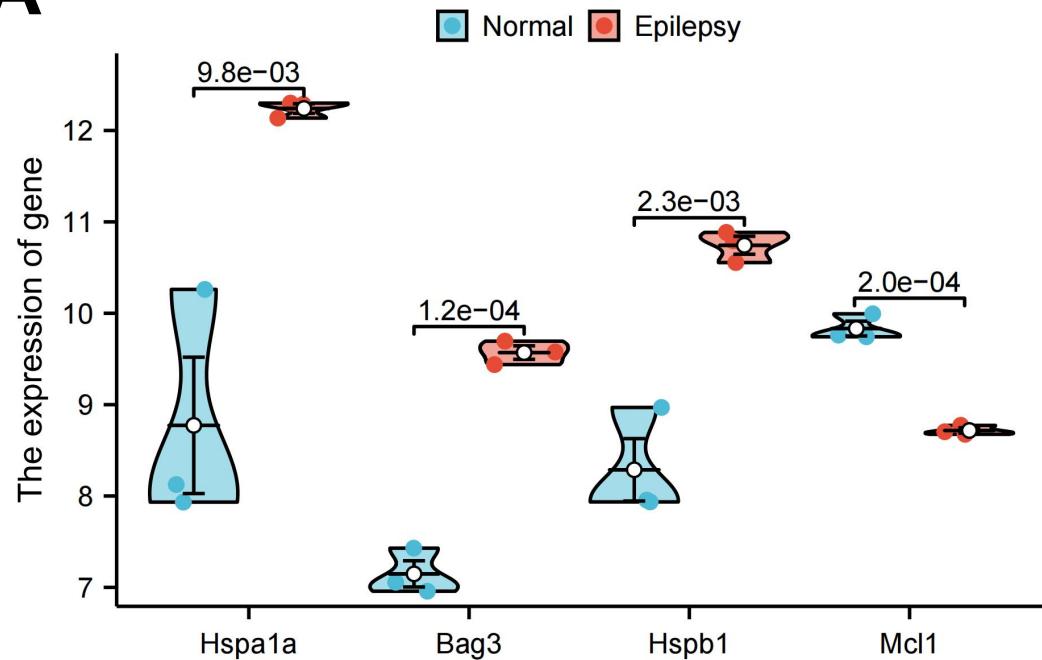**B**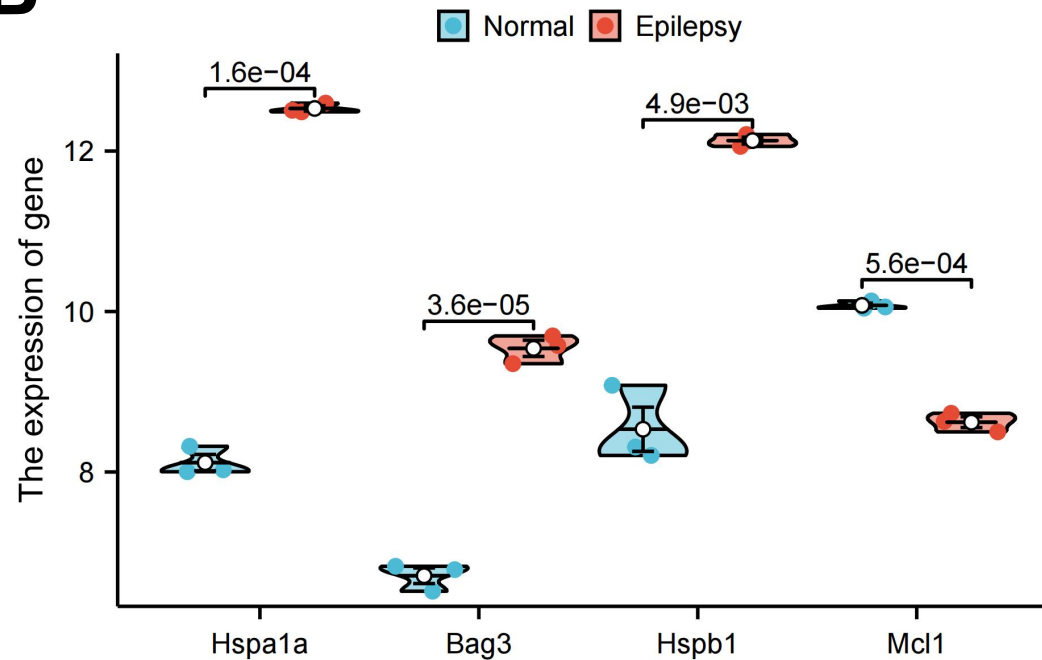

Supplement: Supplementary file 4 — Figure S4. Expression of core genes in normal and epileptic mice. (A) Expression of four core genes in the hippocampus of epileptic mice 6 h post‐injection based on transcriptomic data; (B) Expression of four core genes in the hippocampus of epileptic mice 12 h post‐injection based on transcriptomic data. n = 3. [file CNS-31-e70191-s007.pdf]

**A**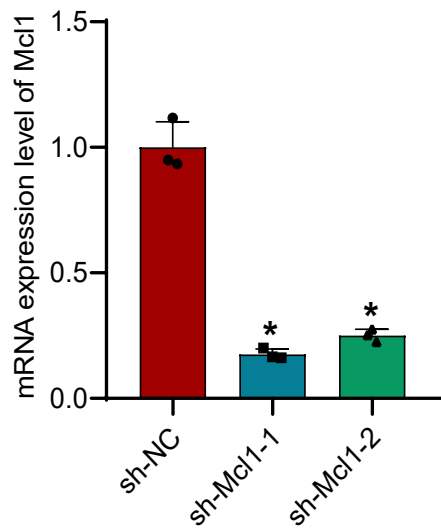**B**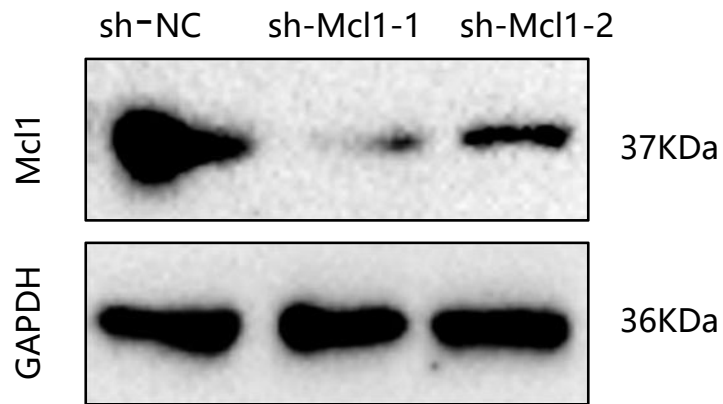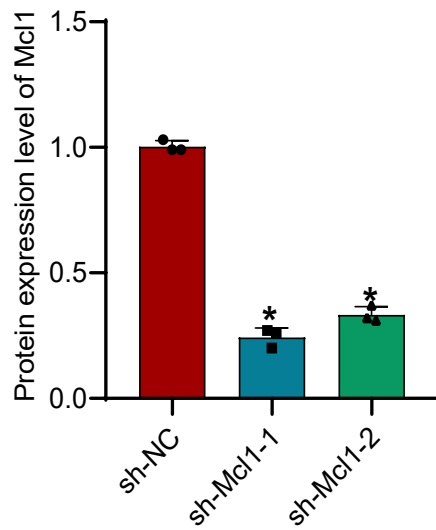

Supplement: Supplementary file 5 — Figure S5. Validation of Lentivirus Silencing Efficiency. (A) Detection of Mcl1 mRNA expression in cells transfected with lentivirus using RT‐qPCR; (B) Evaluation of Mcl1 protein expression in cells transfected with lentivirus through Western Blot experiments. * indicates significance compared to the sh‐NC group, p < 0.05. The cell experiments mentioned were repeated three times. [file CNS-31-e70191-s006.pdf]

**A**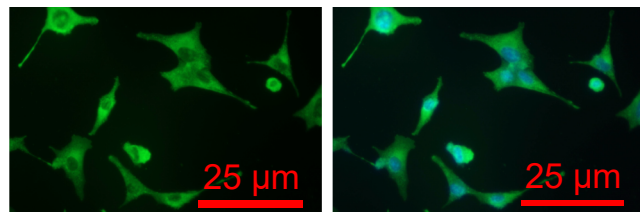**B**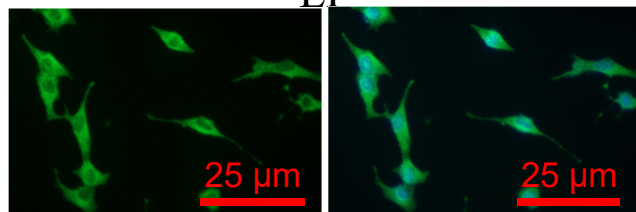**C**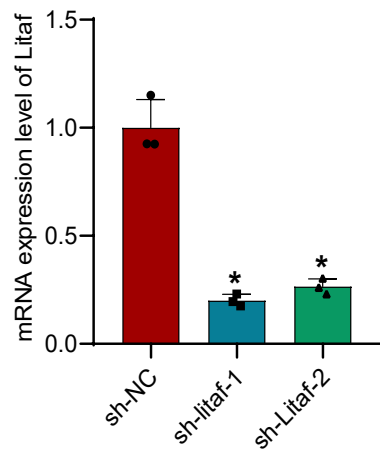**D**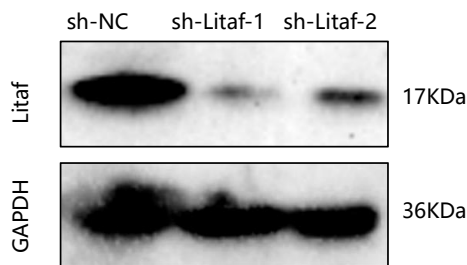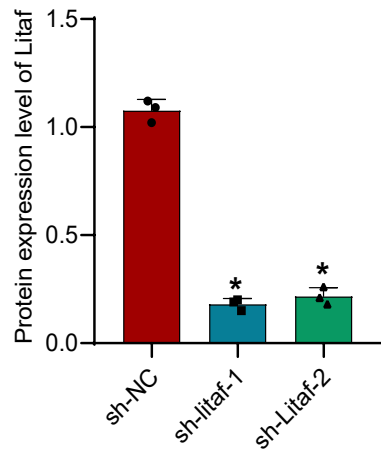

Supplement: Supplementary file 7 — Figure S7. Identification and validation of primary neuronal cells with lentiviral silencing efficiency. (A) Immunofluorescence staining for purity assessment of isolated neuronal cells in the Normal group, where green represents neuronal cells and blue indicates DAPI, scale bar = 25 μm; (B) Immunofluorescence staining for purity assessment of isolated neuronal cells in the EP group, where green represents neuronal cells and blue indicates DAPI, scale bar = 25 μm; (C) RT‐qPCR detecting the mRNA expression of Litaf in cells post lentiviral transfection; (D) Western blot experiment detecting the protein expression of Litaf in cells post lentiviral transfection. * indicates p < 0.05 compared to the sh‐NC group. The cell experiments involved were repeated three times. [file CNS-31-e70191-s002.pdf]
