# Supplementary material for: Impact of LITAF on Mitophagy and Neuronal Damage in Epilepsy via MCL‐1 Ubiquitination
Source: CNS Neurosci Ther. 2025 Jan 7;31(1):e70191. doi: 10.1111/cns.70191 (PMC11705406; doi:10.1111/cns.70191)
Supplement: Supplementary file 6 — Figure S6. Sequence view of the LITAF‐MCL1 interaction. Ub sites are indicated by yellow highlights. [file CNS-31-e70191-s003.pdf]

Statistics

Number of ubiquitination sites	9
Number of potential E3 recogninzing domain	0
Number of potential E3 recogninzing motif	1

Legends

K	Known ubiquitination site
(blue bracket)	Inferred E3 recognizing domain
(black bracket)	Inferred E3 recognizing motif

Notice: Click the lines for details of ubiquitination site and potential E3 recognizing domain/motif.

```
              5         10        15        20        25        30        35        40        45
  1  M F G L K R N A V I G L N L Y C G G A G L G A G S G G A T R P G G R L L A T E K E A S A R R
 47  E I G G G E A G A V I G G S A G A S P P S T L T P D S R R V A R P P P I G A E V P D V T A T
                                                                                    DVT...
 93  P A R L L F F A P T R R A A P L E E M E A P A A D A I M S P E E E L D G Y E P E P L G K R P
     ..L
139  A V L P L L E L V G E S G N N T S T D G S L P S T P P P A E E E E D E L Y R Q S L E I I S R
185  Y L R E Q A T G A K D T K P M G R S G A T S R K A L E T L R R V G D G V Q R N H E T A F Q G
231  M L R K L D I K N E D D V K S L S R V M I H V F S D G V T N W G R I V T L I S F G A F V A K
277  H L K T I N Q E S C I E P L A E S I T D V L V R T K R D W L V K Q R G W D G F V E F F H V E
323  D L E G G I R N V L L A F A G V A G V G A G L A Y L I R
```
