# Supplementary material for: Impact of LITAF on Mitophagy and Neuronal Damage in Epilepsy via MCL‐1 Ubiquitination
Source: CNS Neurosci Ther. 2025 Jan 7;31(1):e70191. doi: 10.1111/cns.70191 (PMC11705406; doi:10.1111/cns.70191)
Supplement: Supplementary file 8 — Table S1. Cell types and marker genes. [file CNS-31-e70191-s001.docx]

**Table S1. Cell Types and Marker Genes.**

| **Cell Type** | **Marker genes** |
| --- | --- |
| Astrocyte | SLC1A2、SLC4A4、DTNA |
| Endothelial Cell | MECOM、FLT1 |
| Microglial Cell | APBB1IP、CD83、P2RY12 |
| Neuron | NRGN、Cux2、MAP1B |
| Oligodendrocyte | MBP、POLR2F、PLEKHH1 |
| Oligodendrocyte Precursor Cell | MEGF11、PCDH15、LHFPL3 |
| Spiral ganglion neuron | ADARB2、GALNTL6、RGS12 |
